# Metabolic Stress and Adaptation in Pancreatic β-Cells to Hypoxia: Mechanisms, Modulators, and Implications for Transplantation

**DOI:** 10.3390/cells14242014

**Published:** 2025-12-17

**Authors:** Jannat Akram, Prianna Menezes, Noorul Ibtesam Idris, Joanna Eliza Thomas, Radwan Darwish, Afrin Tania, Alexandra E. Butler, Abu Saleh Md Moin

**Affiliations:** 1 School of Medicine, Royal College of Surgeons in Ireland-Bahrain, Adliya 15503, Bahrain; 2 Research Department, Royal College of Surgeons in Ireland-Bahrain, Adliya 15503, Bahrain; abutler@rcsi-mub.com

**Keywords:** pancreatic β-cells, hypoxia, mitochondrial dysfunction, β-cell apoptosis, hypoxia-inducible factor-1α (HIF-1α), reactive oxygen species (ROS), islet transplantation, glycolysis, ischemic preconditioning, diabetes pathogenesis

## Abstract

Pancreatic β-cells are metabolically active endocrine cells with a high oxygen demand to sustain glucose-stimulated insulin secretion (GSIS). Hypoxia, arising from vascular disruption, islet isolation, or pathological states such as type 2 diabetes (T2D) and obstructive sleep apnoea (OSA), is a potent metabolic stressor that impairs β-cell function, survival, and differentiation. At the molecular level, hypoxia-inducible factors (HIF-1α and HIF-2α) orchestrate transcriptional programs that shift β-cell metabolism from oxidative phosphorylation to glycolysis, modulate mitochondrial function, and regulate survival pathways such as autophagy and mitophagy. Crosstalk with nutrient-sensing mechanisms, redox regulation, growth factor signaling, and protein synthesis control further shapes adaptive or maladaptive outcomes. Hypoxia alters glucose, lipid, and amino acid metabolism, while mitochondrial dysfunction, oxidative stress, and inflammatory signaling contribute to progressive β-cell failure. Therapeutic strategies including incretin hormones, GABAergic signaling, erythropoietin, ChREBP inhibition, and activation of calcineurin–NFAT or oxygen-binding globins—offer potential to preserve β-cell viability under hypoxia. In islet transplantation, oxygen delivery technologies, ischemic preconditioning, mesenchymal stem cell–derived exosomes, and encapsulation systems show promise in mitigating hypoxic injury and improving graft survival. This review synthesizes current knowledge on β-cell responses to hypoxic stress, with emphasis on metabolic reprogramming, molecular signaling, and translational interventions, underscoring that targeted modulation of β-cell metabolism and oxygen handling can enhance resilience to hypoxia and improve outcomes in diabetes therapy and islet transplantation.

## 1. Introduction

Mammalian cells rely on a steady supply of oxygen and nutrients to produce energy in the form of adenosine 5′-triphosphate (ATP), mainly through mitochondrial oxidative phosphorylation. However, when oxygen levels drop, such as during hypoxia, cells must quickly adapt to survive. Pancreatic β-cells are uniquely hypoxia-sensitive because high metabolic demands, structural diffusion barriers, and insufficient antioxidant defenses together create a precarious oxygen balance. When β-cells are stimulated with high glucose, glycolysis and oxidative phosphorylation markedly increase mitochondrial oxygen consumption, so glucose-stimulated β-cells consume oxygen at rates that can exceed local supply, lowering intracellular oxygen to hypoxic levels even under normoxic conditions [1,2]. Pancreatic β-cells express very low levels of key antioxidant enzymes (glutathione peroxidase, catalase, superoxide dismutase) compared with other islet cells [3], so mitochondrial ROS generated during glucose metabolism overwhelm this defense and make β-cells highly susceptible to hypoxia-associated oxidative damage [4]. Glucose-induced oxygen consumption activates HIF-1α and HIF-2α in β-cells, and hypoxia-mediated HIF-1α activation increases NOX4-derived hydrogen peroxide [5] and induces the transcriptional repressor BHLHE40, which suppresses MAFA, a critical transcription factor for insulin secretion [6]. Acute and chronic hypoxia activate the unfolded protein response (UPR) and endoplasmic reticulum stress in β-cells with upregulation of pro-apoptotic CHOP [7], while hypoxia-driven Ca^2+^ influx causes cytosolic overload partly alleviated by calcium channel blockers, implicating maladaptive UPR signaling and disturbed calcium homeostasis in hypoxia-induced β-cell toxicity [5,8]. The three-dimensional islet architecture and peripheral vascularization impose oxygen diffusion limits that leave many intra-islet β-cells at lower oxygen tension, so islets rapidly become hypoxic after isolation and transplantation, contributing to early non-immune graft failure.

Hypoxia induces several perturbations in pancreatic β-cell metabolism; for instance, insufficient oxygen supply can prompt mitochondrial dysfunction manifested by enhanced mitochondrial fission and increased generation of reactive oxygen species, processes that are mediated by HIF-1α activation [9,10]. Moreover, adaptive responses to hypoxia, such as the upregulation of mitochondrial respiratory complexes, have been observed, which may transiently sustain metabolic function and preserve glucose-stimulated insulin secretion despite a compromised environment [11]. With low oxygen tension, hypoxic islet cells undergo HIF-1α induced anaerobic glycolysis [12]. Pancreatic β-cells can survive hypoxia through utilization of this pathway, with the additional production of lactate, but it comes at the expense of a reduced ability to respond appropriately to high glucose [13]. Nonetheless, if hypoxic stress persists or is severe, these adaptive mechanisms can be overwhelmed, leading to impaired ATP synthesis, dysfunctional insulin secretion, and eventual β-cell demise [10,14]. This reliance on oxygen highlights the metabolic sensitivity of β-cells, as seen in non-diabetic individuals whose pancreatic islet cells exhibit an increase in oxygen consumption following glucose stimulation, a response not observed in pancreatic exocrine cells, which show no significant change in oxygen consumption rate (OCR) under high glucose conditions [15].

In this review article, we aim to assess current knowledge about the effects of hypoxia on pancreatic β-cell metabolism and its adaptation to a limited degree of hypoxia (often associated with activation of HIF-1α). While previous reviews have addressed β-cell responses to hypoxia [16] and the role of HIF signaling in β-cell function [17], this review provides several distinct contributions. First, we systematically integrate recent discoveries on mitochondrial reprogramming, mitophagy regulation, and epigenetic adaptations that were not extensively covered in earlier reviews. Second, we comprehensively synthesize endogenous protective mechanisms (GABA signaling, erythropoietin, incretin hormones, oxygen-binding globins) with exogenous interventions (oxygen-generating biomaterials, MSC-derived exosomes, ischemic preconditioning) to provide a complete translational framework. Third, we critically evaluate emerging technologies, including AI-driven predictive modeling, omics-based discovery platforms, and advanced bioengineering approaches that represent the next generation of therapeutic strategies. Finally, we emphasize clinical relevance to both metabolic diseases (T2D, OSA) and islet transplantation, bridging basic mechanisms with translational applications in a manner not comprehensively addressed previously.

## 2. Cellular Hypoxia

### 2.1. Oxygen-Sensitive Regulation of HIF-1α Expression and Function

**Figure 1:**
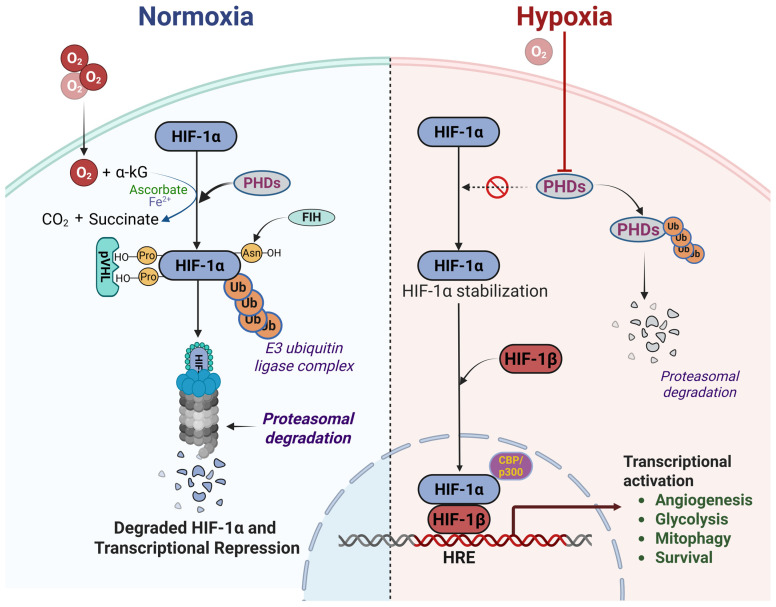
Oxygen-dependent regulation of hypoxia-inducible factor-1α (HIF-1α) in normoxia and hypoxia. **Left** (normoxia): Under normal oxygen tension, HIF-1α is continuously synthesized but rapidly degraded through oxygen-dependent hydroxylation. Prolyl hydroxylase domain enzymes (PHDs) use molecular oxygen, α-ketoglutarate (αKG), Fe^2+^ and ascorbate to hydroxylate specific proline residues within the oxygen-dependent degradation domain of HIF-1α. This modification creates a binding site for the Von Hippel–Lindau tumor suppressor protein (pVHL), the substrate-recognition component of an E3 ubiquitin ligase complex, which ubiquitinates HIF-1α and targets it for proteasomal degradation. In parallel, Factor Inhibiting HIF (FIH) hydroxylates an asparagine residue in the C-terminal transactivation domain of HIF-1α, thereby blocking recruitment of transcriptional co-activators and further repressing HIF-1 activity in normoxia. Together, these hydroxylation events ensure rapid turnover of HIF-1α and transcriptional silencing of HIF-1–responsive genes. **Right** (hypoxia): When oxygen levels fall, PHD activity is inhibited, proline hydroxylation is reduced, and HIF-1α escapes pVHL recognition and ubiquitin-dependent proteasomal degradation. Stabilized HIF-1α accumulates, translocates to the nucleus, and dimerizes with the constitutively expressed HIF-1β subunit to form the active HIF-1 transcription factor complex. This complex binds hypoxia-response elements (HREs) in target gene promoters and recruits co-activators such as CREB-binding protein (CBP) and p300, leading to transcriptional activation of genes that promote angiogenesis, glycolysis, mitophagy, and cell survival. PHD enzymes remain sensitive to co-substrate and cofactor availability; increased αKG, ascorbate, or Fe^2+^ can enhance PHD activity and promote HIF-1α hydroxylation and degradation even at relatively low oxygen tensions, whereas metabolites such as pyruvate and lactate may inhibit PHDs and contribute to a “pseudohypoxic” state. A standard arrowhead, irrespective of its color, usually denotes stimulation or a positive effect. In contrast, a blunt or flat-ended line (┴) generally represents inhibition or a negative effect.

In normoxia, hypoxia-inducible factor-1α (HIF-1α) is tightly controlled by oxygen-dependent hydroxylation. Prolyl hydroxylase domain (PHD) enzymes modify two proline residues within the oxygen-dependent degradation domain of HIF-1α, creating a binding site for the Von Hippel–Lindau tumor suppressor protein (pVHL) (Figure 1) [18]. pVHL serves as the recognition component of an E3 ubiquitin ligase complex that ubiquitinates HIF-1α and targets it for proteasomal degradation via the ubiquitin–proteasome pathway. In parallel, Factor Inhibiting HIF (FIH), an asparaginyl hydroxylase regulated in a similar oxygen-dependent manner, hydroxylates HIF-1α and suppresses its transcriptional activity in normoxia by preventing co-activator recruitment [19,20].

Under hypoxic conditions, PHD activity is inhibited, leading to reduced proline hydroxylation, escape from pVHL recognition, and stabilization of HIF-1α. The stabilized protein accumulates, translocates to the nucleus, and dimerizes with the constitutively expressed HIF-1β subunit, forming the active HIF-1 transcription factor complex that induces genes involved in glycolytic metabolism, angiogenesis, and cell survival. PHDs require multiple co-substrates and cofactors for optimal activity, including molecular oxygen, 2-oxoglutarate (2OG)/α-ketoglutarate (αKG), ferrous iron (Fe^2+^), and ascorbate, and are therefore amenable to regulation not only by O_2_ but also by 2OG availability, oxidative stress, and abnormal levels of endogenous metabolites that mimic 2OG [21]. In this context, pyruvate and lactate have been proposed to promote a “pseudohypoxic” state by inhibiting PHDs, whereas the PHD substrate α-ketoglutarate (αKG), as well as the cofactors ascorbate and Fe^2+^, have been shown to destabilize HIF-1α in a dose-dependent manner even under low oxygen conditions. For example, αKG can increase PHD affinity for oxygen and thereby promote HIF-1α hydroxylation and degradation at oxygen tensions that would otherwise permit its stabilization [22].

### 2.2. Hypoxia and HIF in Pancreatic β-Cells

β-cells consume more oxygen than other types of cells on average and are particularly vulnerable to declining oxygen availability. HIF-1α plays a crucial role in regulating pancreatic β-cell function and differentiation. Under normal oxygen conditions, HIF-1α is present at low levels in β-cells and is essential for glucose-stimulated ATP generation and insulin secretion [23,24]. However, chronic activation of HIF-1α in hypoxic conditions impairs β-cell function by shifting metabolism from aerobic to anaerobic glycolysis, disrupting glucose-stimulated insulin secretion [17,25]. During pancreatic development, HIF-1α levels are initially high and decrease over time, with low oxygen tension inhibiting β-cell differentiation through HIF-1α-mediated repression of Neurogenin3 expression [26,27]. Research suggests that hypoxia in pancreatic β-cells contributes to the progression of type 2 diabetes (T2D) by impairing insulin secretion and promoting cell loss [28]. In T2D, islet amyloid derived from islet amyloid polypeptide (IAPP) forms toxic oligomers, potentially causing β-cell death and dysfunction [29]. IAPP-induced misfolded protein stress activates HIF1α/PFKFB3 signaling, leading to increased glycolysis and mitochondrial changes that protect against toxic oligomer stress but compromise β-cell function [30]. Metabolic stressors like insulin resistance and hyperglycemia may exacerbate amyloid formation by increasing IAPP production and impairing prohormone processing [31]. This creates a feed-forward cycle of β-cell decline, where reduced β-cell mass increases metabolic demand on remaining cells, further promoting dysfunction and amyloid formation [31].

### 2.3. β-Cell Metabolic Aging: FOXO1–HIF Cross Talk and the Roles of HIF-1 and HIF-2

FOXO1 is a central β-cell identity factor that integrates nutrient and stress signals with transcriptional programs maintaining insulin production, oxidative metabolism, and resistance to cellular stress [32,33]. Loss of FOXO1 activity in mouse β-cells leads to dedifferentiation into progenitor-like and α-like cells with re-expression of NGN3, Oct4, Nanog and ALDH1A3 and loss of Pdx1, MafA and Nkx6.1, establishing FOXO1 as a gatekeeper against β-to-non-β lineage drift, and human T2D islets show similar patterns with reduced FOXO1 and β-cell transcription factors, increased *ALDH1A3* and “disallowed” genes such as *LDHA* and *MCT1*, and a shift toward hormone-negative or α-like endocrine cells [32,34,35,36]. Hypoxia and chronic metabolic stress activate HIF-1α in β-cells, reprogramming glucose metabolism toward glycolysis and away from mitochondrial oxidative phosphorylation and thereby attenuating ATP generation needed for glucose-stimulated insulin secretion [2,37]. Experimental HIF-1α stabilization in human islets and mouse β-cell lines decreases insulin content and secretion while upregulating β-cell “disallowed” genes (*GLUT1*, *LDHA*, *NGN3*, *ALDH1A3*), directly linking sustained HIF-1α activity to hypoxia-induced β-cell dedifferentiation [27]. In parallel, chronic hypoxia induces transcriptional repressors such as BHLHE40 and ATF3 downstream of HIF signaling, which suppress MafA, PGC-1α, insulin genes and IRS2, further weakening oxidative metabolism, exocytotic machinery and insulin gene expression in β-cells and reinforcing a FOXO1-linked dedifferentiation and metabolic aging phenotype [6].

**Figure 2:**
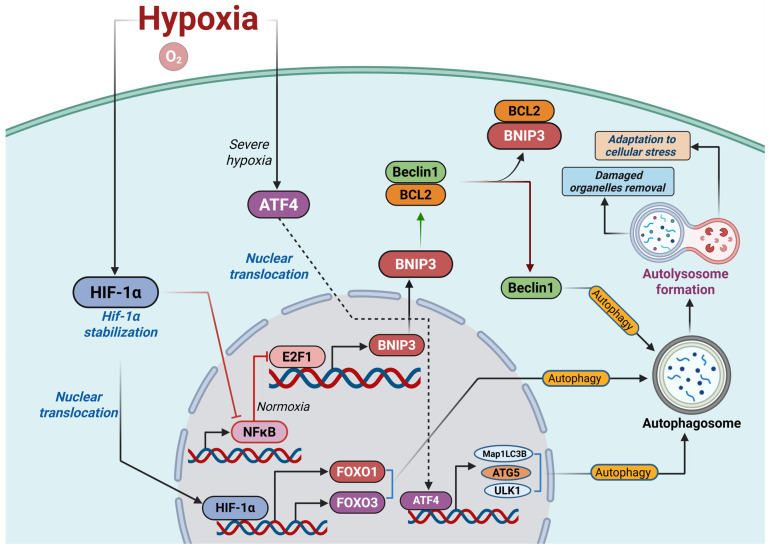
Hypoxia–autophagy transcriptional network. Under hypoxic stress, several transcription factors cooperate with or substitute for FOXO1 to induce autophagy. Moderate hypoxia stabilizes hypoxia-inducible factor-1α (HIF-1α), which translocates to the nucleus and directly upregulates the mitophagy receptors BCL2/adenovirus E1B 19 kDa protein-interacting protein 3 (BNIP3) and BNIP3-like protein (BNIP3L/NIX), establishing a canonical HIF–BNIP3 axis that links oxygen sensing to mitochondrial clearance. With more severe hypoxia, activating transcription factor-4 (ATF4) becomes dominant, driving nuclear transcription of BNIP3 and core autophagy genes, including microtubule-associated proteins 1A/1B light chain 3B (*MAP1LC3B*), autophagy-related 5 (*ATG5*) and Unc-51-like autophagy activating kinase 1 (*ULK1*). The E2F transcription factor 1 (E2F1) and nuclear factor kappa-light-chain-enhancer of activated B cells (NF-κB) oppositely regulate BNIP3 expression: in normoxia, NF-κB restrains E2F1 binding at the BNIP3 promoter, whereas hypoxia relieves this inhibition, permitting E2F1-driven BNIP3 transcription and coordinated activation of *ULK1*, *MAP1LC3B* and *ATG5*. BNIP3 also promotes autophagy by disrupting the inhibitory complex between B-cell lymphoma 2 (BCL-2) and Beclin-1 (BECN1), thereby freeing BECN1 to initiate autophagosome formation. Forkhead box proteins FOXO1 and FOXO3 further contribute to hypoxia-induced autophagy, reinforcing the broader FOXO family’s role in oxygen-stress adaptation. The resulting autophagosomes and autolysosomes mediate the removal of damaged organelles and contribute to cellular stress adaptation. Mechanisms depicted are derived predominantly from non–β-cell models and are shown here as a proposed framework that has yet to be systematically validated in pancreatic β-cells. A standard arrowhead, irrespective of its color, usually denotes stimulation or a positive effect. In contrast, a blunt or flat-ended line (┴) generally represents inhibition or a negative effect.

A central node linking hypoxic signaling to metabolic aging in human β-cells is the HIF-1α–FOXO1 axis, which coordinates autophagy and broader stress-adaptation programs. Under acute hypoxia, HIF-1α induces FOXO1 together with the autophagy markers LC3 and p62/SQSTM1 in human islets, and FOXO1-dependent autophagy (Figure 2) constrains apoptosis, indicating that HIF-1α-driven FOXO1 activation initially serves as a pro-survival response that helps maintain β-cell mass under oxygen stress [38]. In this setting, pharmacologic or genetic inhibition of autophagy or FOXO1 exacerbates β-cell death, whereas in T2D pancreata autophagy markers are diminished and inversely associated with HbA1c, consistent with a progressive breakdown of HIF-1α–FOXO1–autophagy signaling as metabolic disease advances [33,39]. Since autophagy is essential for β-cell organelle quality control, insulin granule turnover, and adaptive remodeling under nutrient stress, impaired FOXO1-regulated autophagy provides a plausible mechanistic link by which chronic hypoxia accelerates β-cell metabolic aging. FOXO1 itself is a classical “longevity” factor whose activity is modulated by acetylation, phosphorylation, and nuclear–cytoplasmic shuttling in response to oxidative stress, nutrient cues, and insulin/IGF-1 signaling [40]. In β-cells, deacetylated FOXO1 promotes mitochondrial fitness by restraining fatty acid oxidation and mitigating lipotoxic stress, implying that appropriately tuned FOXO1 activation preserves youthful mitochondrial function and glucose responsiveness [41]. Given that both hypoxia and glucolipotoxicity converge on ROS generation and stress-activated kinase pathways, prolonged HIF-1α activation, oxidative stress, and FOXO1 post-translational modification are likely to cooperate in shifting FOXO1 from a protective, pro-autophagic state toward loss of β-cell identity and dedifferentiation.

HIF-2α introduces an additional regulatory layer into this cross-talk by preferentially safeguarding mitochondrial integrity and redox homeostasis in metabolically stressed β-cells. In obesity, metabolic stress induces β-cell HIF-2α, which upregulates antioxidant genes such as *Sod2* and *Cat*, limits mitochondrial ROS, and preserves both mitochondrial mass and glucose-stimulated insulin secretion; conversely, β-cell-specific HIF-2α deletion worsens mitochondrial damage and glucose intolerance under a high-fat diet [42]. Together, these findings support a model in which HIF-2α activation constitutes an early compensatory program that counteracts β-cell metabolic aging, whereas excessive or sustained HIF-1α activity in the same cells promotes glycolytic reprogramming, oxidative injury, and FOXO1-linked erosion of β-cell identity.

### 2.4. Hypoxia–Autophagy Transcriptional Control Beyond FOXO1

Multiple transcription factors cooperate with or substitute for FOXO1 to drive autophagy under hypoxia (Figure 2). HIF-1α directly induces the mitophagy adaptors BNIP3 and BNIP3L (NIX), establishing a canonical HIF–BNIP3 axis for hypoxia-evoked autophagy [43]. The depth of oxygen deprivation appears to gate which factors dominate: moderate hypoxia favors HIF-1α-dependent BNIP3 activation, whereas severe hypoxia shifts control to activating transcription factor-4 (ATF4) [44]. ATF4 transcriptionally upregulates MAP1LC3B by binding a cAMP-response element in its promoter, and more broadly enhances essential autophagy genes under hypoxia [45,46]. E2F1 and NF-κB oppositely tune BNIP3 expression—NF-κB restrains E2F1 binding at the BNIP3 promoter in normoxia, while hypoxia relieves this brake to permit E2F1-driven BNIP3 transcription [47]. Beyond BNIP3, E2F1 directly targets ULK1, MAP1LC3, and BNIP3 and indirectly regulates ATG5, extending its reach across the autophagy initiation and elongation modules [48]. Beclin-1 (BECN1) is a shared target of E2F1 and NF-κB, and BNIP3 promotes autophagy by releasing BECN1 from BCL-2-mediated inhibition [47]. FOXO3 also contributes to hypoxia-induced autophagy, reinforcing the broader FOXO family’s role in oxygen-stress adaptation [49]. It is important to note that these hypoxia–autophagy transcriptional mechanisms have been delineated primarily in non–β-cell models—including fibroblasts, mouse embryonic fibroblasts and a range of human cancer cell lines (colon, breast, prostate, glioblastoma, hepatocellular and head and neck tumors)—and have not yet been systematically validated in pancreatic β-cells.

## 3. Metabolic Pathways and β-Cell Function Under Normoxia

### 3.1. Glucose Sensing

Shortly after eating, circulating levels of nutrients such as glucose, amino acids, peptides, and lipids rise, prompting pancreatic β-cells to secrete insulin in proportion to plasma glucose levels—thereby promoting nutrient uptake and simultaneously suppressing their release from organs like the liver [50]. GLUT2 is the primary glucose transporter in pancreatic β-cells, enabling rapid, insulin-independent glucose equilibration via facilitated diffusion. Its low affinity (high Km) ensures efficient uptake at high glucose levels [51]. In intact islets, glucose transport capacity far exceeds glycolytic demand, allowing intra- and extracellular glucose levels to equalize within seconds. Only a >90% reduction in transport significantly impairs glucose metabolism [52]. Pancreatic β-cells sense glucose primarily through the activity of glucokinase, a key enzyme that catalyzes the initial and rate-limiting step of glucose metabolism [53,54]. Glucokinase exhibits distinct kinetic properties, namely a low affinity and high catalytic capacity for glucose, that enable β-cells to respond dynamically across a wide range of blood glucose concentrations and to initiate a cascade of metabolic events culminating in insulin secretion [53,55]. The phosphorylation of glucose by glucokinase drives glycolysis, thereby increasing the production of ATP via mitochondrial oxidative phosphorylation; the rise in the ATP/ADP ratio in turn leads to the closure of ATP-sensitive K^+^ channels, resulting in cell membrane depolarization, preventing glucose-exit from cells through GLUT2, opening of voltage-dependent Ca^2+^ channels, and ultimately insulin granule exocytosis [55,56]. Morphological studies have also demonstrated that changes in glucokinase distribution preceded alterations in insulin localization within beta cells, further underscoring its central role as a glucose sensor [57].

### 3.2. Mitochondrial Oxidation

Mitochondrial metabolism is central to both the triggering and amplifying phases of glucose-stimulated insulin secretion (GSIS) in pancreatic β-cells, primarily through ATP production via the electron transport chain (ETC) complexes I–V [58]. Upon stimulation by glucose, the flow of electrons toward the respiratory chain elevates ATP production by inducing mitochondrial membrane hyperpolarization, a process that initiates the cascade leading to insulin secretion [59]. A recent study emphasizes that calcium uptake into mitochondria is essential for optimal GSIS. Inhibition of sodium-calcium exchangers reduces mitochondrial calcium levels, highlighting the importance of effective metabolic coupling during nutrient excess [60]. The production of NADH is critical in this context, as it’s a key substrate for ATP synthesis, facilitated by the transfer from cytosolic NADH to mitochondria via NADH shuttles [61]. The glycerol phosphate and malate-aspartate shuttles play pivotal roles here, with the former delivering electrons to coenzyme Q (complex II) in the ETC, while the latter involves the regeneration of NAD+, crucial for maintaining the redox balance required for aerobic metabolism and insulin secretion [61,62].

Glutamate dehydrogenase (GDH-1) supports insulin secretion by converting glutamate to α-ketoglutarate, enhancing metabolic flexibility, especially during hypoglycemia. Elevated ADP activates GDH-1, sustaining ATP production by regulating the intramitochondrial NADH/NAD+ ratio during energy stress [62]. Recent investigations have unveiled the role of KATP channels, where mitochondrial ATP production governs their activity and thus regulates both insulin secretion and the cellular calcium oscillations essential for this process [59]. Moreover, the interaction between mitochondrial dynamics and insulin signaling further stresses the need for intact mitochondrial function in β cells. Disruption of mitochondrial processes can lead to increased reactive oxygen species (ROS) production, aggravating metabolic dysfunction and impairing the β cells’ ability to secrete insulin effectively [59]. This reflects broader metabolic consequences and underlines mitochondrial bioenergetics as an attractive target for therapeutic strategies aimed at enhancing insulin secretion and countering diabetes-related pathologies [58,62].

### 3.3. Cellular Metabolite—Glutamate Enhances β-Cell Function

The additional product of glucose metabolism, glutamate, contributes to the amplification pathway of calcium signaling in pancreatic β-cells [63,64]. Intracellular glutamate can enhance insulin secretion [63,64], while extracellular glutamate can activate ionotropic receptors that may inhibit insulin exocytosis [63] (note that some studies show a more complex relationship, with glutamate acting as both an enhancer and inhibitor depending on the context) [65]. Consequently, glutamate serves dual roles in β-cells, acting as an amplifying signal and a potential negative feedback regulator [63]. The mitochondrial enzyme glutamate dehydrogenase (GDH), encoded by the GLUD1 gene, mediates the reversible reaction: α-ketoglutarate + NH_3_ + NADH ↔ glutamate + NAD+ [66,67]. Furthermore, leucine, along with adenine and guanine nucleotides, has been demonstrated to play allosteric regulatory roles in modulating GDH activity in eukaryotic cells [67,68]. GDH can operate in an anaplerotic manner, generating α-ketoglutarate to support the tricarboxylic acid (TCA) cycle, or in a cataplerotic manner, producing glutamate at the expense of α-ketoglutarate [66,69]. Thus, when GDH is allosterically activated, it may serve as an amino acid sensor that triggers insulin release in response to glutamine stimulation or may participate in a glucose-induced amplification pathway by generating glutamate [63,70].

## 4. Hypoxia-Related Metabolic Stress and Mitochondrial Dysfunction in Pancreatic β-Cells

### 4.1. Hypoxia in Obstructive Sleep Apnoea (OSA): Impact on β-Cell Function and Insulin Maturation

There is a high demand for oxygen consumption in pancreatic β-cells due to mitochondrial respiration. Hypoxia is defined as reduced levels of oxygen that cause reduced functioning. Pancreatic β-cells are extremely sensitive to hypoxia due to their oxygen dependence [7]. The development of prediabetes and ensuing T2D is influenced by both an increase in insulin resistance and a deterioration in β-cell function [71]. Obstructive sleep apnoea (OSA) is a chronic, sleep-related respiratory disorder that affects an increasing number of people. Periodic constriction and occlusion of the pharyngeal airway during sleep are symptoms of OSA [72]. OSA causes increased negative intrathoracic pressure, fragmented sleep, and brief episodes of sleep hypoxia during sleep [73]. Obesity is a risk factor for OSA, and its prevalence is rising globally [74]. Chronic intermittent hypoxemia seen in OSA has been linked to the onset of insulin resistance and pancreatic β-cell dysfunction [75]. In mice with diet-induced obesity as well as mice with genetic obesity, chronic intermittent hypoxia worsened fasting hyperglycaemia, glucose intolerance and insulin resistance [76,77].

Research shows OSA causes amplification of sympathetic activity in obese patients. Hypoxia in OSA may activate the sympathetic nervous system (SNS) via peripheral chemoreceptors [78]. Oxidative stress, increased HIF-1α signaling and decreased HIF-2 signaling, as well as increased endothelin-1, have been proposed as key mechanisms in intermittent hypoxia-induced SNS activation [79]. Insulin resistance is facilitated by the release of adrenal epinephrine during sympathetic activation, which causes glucose synthesis and reduces insulin secretion [80]. After the onset of sleep, nocturnal glucose levels tend to rise in people with moderate to severe OSA [81]. Bialasiewicz et al. showed that, in patients with mild or moderate OSA, the normal trend towards lower glucose levels during REM sleep was reversed during apnoeic events [82].

**Figure 3:**
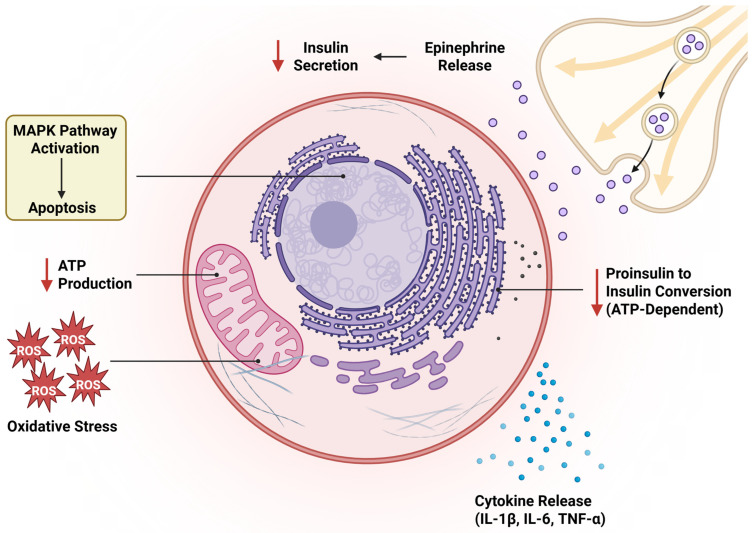
Hypoxia disrupts pancreatic β-cell metabolism, insulin processing and survival through mitochondrial dysfunction, oxidative stress and inflammation.

Insulin is first translated as pre-proinsulin and, in the endoplasmic reticulum (ER), is cleaved to proinsulin. Prohormone convertases-2 (SPC2), PC3/PC1 (SPC3), and carboxypeptidase E then promote the maturation of proinsulin into insulin, which takes place in the secretory granules [83]. Granule pH and Ca^2+^ concentration control SPC function. Granule pH and Ca^2+^ concentration regulation is an ATP-dependent activity. Changes in ion channel conductance, alterations in membrane potential, and elevations in intracellular calcium are among the direct impacts of hypoxia upon the β-cell. Furthermore, hypoxia causes activation of transcription factors due to increases in intracellular calcium. The hypoxic stress of OSA includes brief episodes of tissue hypoxia followed by brief intervals of tissue reperfusion. It has been proposed that the oxidative damage caused by ischemia-reperfusion during sleep-disturbed breathing inhibits β-cell ATP synthesis, which lowers pro-insulin conversion and hence lowers insulin levels [84]. Inflammatory cytokines are increased in OSA patients [85,86,87]. Intermittent hypoxia increases cytokines and inflammatory mediators like interleukin-1 (IL-1), IL-1α, IL-4, IL-6 and IL-13 [88]. Elevated inflammatory mediators can worsen systemic or local inflammation. As a result of the oxidative stress brought on by intermittent hypoxia in OSA, inflammatory cytokines and vasoactive chemicals are released, which may lead to endothelial damage. Increased cytokine levels and systemic inflammation have been associated with increased insulin resistance [89]. Wang et al. demonstrated that, depending on the oxygen concentration, chronic intermittent hypoxia (CIH) increased the production of TNF-a, IL-1β and IL-6, as well as activated members of the mitogen-activated protein kinase (MAPK) family, extracellular regulated protein kinase (ERK), c-Jun N-terminal kinase (JNK) and p38. Through the MAPK signaling route, CIH disrupted insulin secretion and induced inflammation and cell death in pancreatic tissue [90] (Figure 3).

A significant characteristic of islets in T2D is pancreatic β-cell apoptosis. The intrinsic or Bcl-2-regulated pathway and the extrinsic or death receptor-mediated pathway are the two principal pathways through which apoptosis or “programmed cell death” takes place. The intrinsic route is controlled by the balance between the pro- and anti-apoptotic Bcl-2 family members, while the extrinsic route is mediated by the Tumor Necrosis Factor (TNF) superfamily, such as FasL, which binds to cell-surface death receptors like Fas or TNFR [91].

### 4.2. Hypoxia-Induced Mitochondrial Dysfunction and Oxidative Stress in Pancreatic β-Cells

The pancreatic β-cell is highly dependent on oxygen because of its intense metabolic activity. Metabolism of glucose in the mitochondria provides energy for the synthesis of insulin as well as for the insulin secretion signaling pathway, the main function of the β-cell. Changes in mitochondrial function are expected to occur in hypoxia-induced loss of function. ROS-induced metabolic stress results in mitochondrial dysfunction. In pancreatic β-cells of conditional mitochondrial matrix targeting mitophagy reporter (CMMR) mice, hypoxia increases mitochondrial ROS [92,93,94], leading to reduced mitochondrial membrane potential, decreased ATP production, and impaired glucose-stimulated insulin secretion (GSIS) [95]. Apoptotic markers like cleaved caspase-3 are also upregulated. Mechanistically, hypoxia limits electron flow at ETC complexes I and III, causing one-electron leakage to oxygen and generating superoxide radicals, key contributors to β-cell mitochondrial dysfunction and death [96,97,98,99,100,101]. Further, to produce energy, mitochondria require oxygen. However, a process called proton leakage occurs via two distinct routes. First, basal leak through the inner mitochondrial membrane accounts for ~20–30% of oxygen consumption, as a result of passive diffusion and carrier slippage [102,103]. Second, β-cells express uncoupling protein 2 (UCP2), which mediates regulated proton leak in response to elevated mitochondrial superoxide [104,105,106]. UCP2 activation decreases the proton gradient, lowers ATP synthesis efficiency, and impairs GSIS. Knockdown or inhibition of UCP2 in β-cells improves ATP production and restores insulin secretion [105,107]. Uncharged molecules with an unpaired valence electron are known as free radicals [108]. Continuously increasing glycolytic flux may potentially enhance ROS production in the β-cell when glucose clearance begins to be compromised due to peripheral insulin resistance, with potentially serious pathological repercussions. Increases in intracellular Ca^2+^ drive the production of ROS by the mitochondria and activate protein kinase C, which, in turn, causes the production of superoxide and other species that are nicotinamide adenine dinucleotide phosphate (NADPH) oxidase-dependent [16]. The term “mitochondrial uncoupling” describes the dissociation of electron-dependent oxygen consumption from ATP production within the respiratory chain [108]. Small, intramembranous mitochondrial proteins called uncoupling proteins (UCPs) are essential for thermogenesis and are tissue-specific [109]. UCP2 is a mitochondrial anion carrier that dissipates the proton gradient created across the mitochondrial inner membrane in order to uncouple the synthesis of ATP from oxidative phosphorylation [110]. There is increasing evidence that UCP2 helps to regulate the formation of ROS that are produced by mitochondria [106,111]; this might offer a crucial mechanism to regulate ROS produced by the mitochondria that control cellular activity and/or to avert oxidative stress, which is brought on by continuous ROS build-up and eventually results in oxidative damage and cytotoxicity. NADPH oxidases (NOX) are the main plasma membrane or cytosolic superoxide generators among the cytosolic ROS sources in pancreatic cells [7]. These cells use NADPH as an electron donor and catalyse the one-electron reduction of oxygen to produce O^2−^. NOX2 is an isoform of NADPH [112]. NOX2 functions as a suppressor of the secretory response in pancreatic β-cells by lowering cAMP secondary to ROS production [113].

Impaired β-cell mitochondrial metabolism in people with mitochondrial deoxyribonucleic acid (mtDNA) mutations may predispose them to β-cell malfunction [114]. A connection may exist between mtDNA mutations and ROS production, as mtDNA mutations may encourage ROS production, further compromising mitochondrial function. MtDNA is more vulnerable to oxidative damage than nuclear DNA because of its proximity to high oxygen consumption, the lack of protective histones and limited DNA repair mechanisms [115]. However, this apparent connection is not supported by any in vivo evidence as yet. Of note, despite accelerated aging, transgenic mice that spontaneously accumulate mtDNA mutations do not show greater ROS production [91].

## 5. Adaptive and Protective Responses to Hypoxia in Pancreatic β-Cells

### 5.1. HIF-1α–Mediated Islet Angiogenesis in Hypoxic Environments

During pancreatic islet transplantation, the engrafted islets initially experience a hypoxic environment due to disruption of their native vasculature, which necessitates rapid revascularization to ensure graft survival and function [116,117]. Under these hypoxic conditions, the oxygen-dependent degradation of hypoxia-inducible factor 1 alpha (HIF-1α) is inhibited, allowing its stabilization and translocation into the nucleus [118]. Once in the nucleus, HIF-1α dimerizes with HIF-1β and binds to hypoxia-responsive elements (HREs) in the promoter regions of several genes that are pivotal to angiogenesis [119,120]. A key target of HIF-1α is the vascular endothelial growth factor (VEGF), a potent angiogenic cytokine that promotes endothelial cell proliferation, migration, and new vessel formation [121]. HIF-1α-driven VEGF expression is central to establishing a pro-angiogenic microenvironment necessary for the revascularization of transplanted pancreatic islets [122,123]. Furthermore, angiogenic gene products such as Notch1 are also upregulated in response to HIF-1α activation and contribute to the proper maturation and sprouting of new vessels, optimizing blood flow and nutrient supply to the islet grafts [124]. Recent studies have highlighted strategies to harness this pathway for improving transplant outcomes. For example, pharmacological modulation using agents like liraglutide has been shown to enhance HIF-1α expression in pancreatic islets, thereby promoting angiogenesis and improving graft viability [125]. Similarly, other studies have indicated that the induction of HIF-1α not only facilitates revascularization but also provides cytoprotective effects on β-cells, enhancing their survival during the critical post-transplant period [122]. Consequently, the HIF-1α-mediated angiogenic cascade forms an essential adaptive response, supplying the transplanted islets with a renewed vascular network that supports oxygenation, nutrient delivery, and overall graft integration into the host tissue.

### 5.2. Maternal–Fetal Hypoxia Programming and Neonatal Islet Adaptations

Maternal metabolic conditions during gestation significantly influence the fetal oxygen and nutrient environment, resulting in lasting effects on islet development and postnatal function [126]. These findings indicate that maternal stressors that influence placental and fetal hypoxia pathways can epigenetically influence the endocrine pancreas, wherein they increase susceptibility to β-cell dysfunction and diabetes later in life [127]. The oxygen supply remains a critical determinant for the survival and function of islet tissue, with several recent studies elucidating the distinct metabolic adaptations of neonatal islets compared to their adult counterparts under hypoxic conditions. Early work demonstrated that neonatal rat islet cells display higher tolerance to low oxygen tensions, with increased production of pyruvate and lactate, which confers them greater metabolic flexibility relative to adult islet cells that primarily rely on lactate production [128]. This metabolic plasticity is understood to be accompanied by differential regulation of key β-cell transcription factors and metabolic enzymes under hypoxic stress, as evidenced by altered gene expression patterns in hypoxia-exposed β-cells [129]. Neonatal β-cells exhibit poor glucose-induced insulin secretion due to immature metabolic pathways, including reduced expression of key mitochondrial shuttles and enzymes such as pyruvate carboxylase and carnitine palmitoyl transferase 2. This metabolic immaturity, persisting even at 28 days, underlies their delayed acquisition of glucose responsiveness [130]. Such differences in cellular metabolism explain why neonatal islets continue to maintain their baseline insulin secretory response even under severe hypoxia, whereas adult islets show a marked decline in glucose-stimulated insulin secretion.

#### 5.2.1. Maternal Metabolic Stress, Placental HIFs, and Fetal β-Cell Programming

The intrinsic developmental adaptation to hypoxia, maternal metabolic conditions during gestation, significantly influence the fetal oxygen and nutrient environment. This results in lasting effects on islet development and postnatal function [126]. Factors such as maternal undernutrition, pre-existing diabetes and obesity can cause metabolic and oxidative stress on the placenta, causing a disruption to the nutrient and oxygen delivery to the fetus [131]. These stressors are known to influence placental expression of hypoxia-inducible factors, especially HIF-1a, and thereby modify downstream angiogenic and metabolic pathways that regulate fetal growth [132]. Dysregulated placental HIF signaling can result in disruption of the intrauterine oxygen tension and nutrient constitution, which will be sensed by the developing fetal pancreas, and thereby influence β-cell proliferation, differentiation, and maturation [133]. Experimental models demonstrate that ongoing maternal hypoxia or hyperglycemia can disrupt β-cell lineage specification and mitochondrial function. This reduces insulin gene expression and blunt glucose responsiveness in the offspring, which is consistent with developmental “mis-programming” of β-cell function [127].

Notably, this HIF-mediated programming may act through epigenetic mechanisms that fix mismatched β-cell gene expression patterns beyond gestation [134]. Abnormal stabilization of HIF-1α in the placenta or fetal pancreas has been associated with changes in the chromatin structure and DNA methylation of important β-cell transcriptional regulators such as *Pdx1*, *Mafa*, and *Glut2*. This results in persistent deficits in insulin’s secretory ability and metabolic resilience. These findings indicate that maternal stressors that influence placental and fetal hypoxia pathways can epigenetically influence the endocrine pancreas, wherein they increase susceptibility to β-cell dysfunction and diabetes later in life [127].

#### 5.2.2. Intrinsic Biological Adaptations of Neonatal Islets to Hypoxia

The superior hypoxia tolerance of neonatal islets reflects intrinsic biological adaptations that distinguish them from mature adult islets. In neonatal rat islets, hypoxia causes a smaller fall in ATP and cAMP, and pyruvate levels remain higher than in adult islets, while both pyruvate and lactate are actively produced—indicating enhanced metabolic flexibility to use available oxygen yet maintain glycolytic ATP production when oxidative phosphorylation is compromised [128,135]. Consistently, neonatal porcine islets are more resistant to hypoxia-induced apoptosis than adult porcine islets, further underscoring the intrinsic hypoxia tolerance of neonatal islet tissue [136]. This elevated glycolytic capacity is accompanied by LDH-dependent lactate and pyruvate handling consistent with active anaerobic glycolysis in neonatal islets, and LDH-mediated regeneration of NAD+ sustains glycolytic flux under oxygen limitation. In parallel, neonatal islets maintain ATP levels and insulin secretion at lower oxygen tensions than adult islets, indicating a glycolysis-driven adaptation that confers greater resistance to hypoxia-induced dysfunction and cell death [128,136,137]. At the mitochondrial level, neonatal β-cells display a metabolically immature profile compared with adult β-cells. Transcriptomic and proteomic studies show that neonatal islets have reduced expression of genes involved in mitochondrial shuttles and oxidative metabolism, with oxidative phosphorylation networks becoming more prominent as β-cells mature [130]. Additionally, mitochondrial quality-control pathways are critical for neonatal β-cell survival and maturation. In two-week-old mice, loss of the lysosomal cholesterol transporter NPC1 blocks autophagic and mitophagic flux, leading to accumulation of autophagosomes/lysosomes, impaired mitochondrial respiration, and increased neonatal β-cell apoptosis and defective postnatal β-cell differentiation [138]. In neonatal porcine islets, induction of autophagy by human mesenchymal stem cell–derived conditioned medium protects against hypoxia-induced cell death, underscoring the cytoprotective role of mitochondrial quality control in immature islets. More broadly, β-cell–specific studies show that mitophagy machinery such as the CLEC16A–NRDP1–USP8 complex removes damaged mitochondria and limits apoptotic β-cell loss during inflammatory and metabolic stress [139]. The transcriptional profile of neonatal β-cells is skewed toward a glycolytic, immature metabolic program. Compared with fully mature adult β-cells, neonatal and other immature β-cell states retain higher expression of ‘disallowed’ glycolytic genes such as *hexokinase 2* and *lactate dehydrogenase A*, which are progressively silenced as β-cells mature. In human islets, glucose transporters including GLUT1 are already prominently expressed in fetal and neonatal β-cells, with GLUT2 relatively enriched earlier in development before shifting towards the adult pattern [140]. Many of these glycolytic and transport genes are canonical hypoxia-inducible factor-1α (HIF-1α) targets in other tissues, suggesting that neonatal β-cells are transcriptionally primed to rely on glycolytic ATP production and may better tolerate transient limitations in oxygen availability, even though direct evidence for globally increased HIF activity at baseline in neonatal islets is still limited. Moreover, survival of neonatal β-cells during the postnatal remodeling phase is tightly controlled by Bcl-2 family proteins. Developmental studies show dynamic changes in the expression of anti-apoptotic members such as Bcl-2/Bcl and pro-apoptotic factors including Bak and Bax across fetal, neonatal and adult life, coinciding with waves of β-cell apoptosis and remodeling of islet mass [141]. Experimental upregulation of Bcl-2 in neonatal rat islets—for example, by ciliary neurotrophic factor or growth factor signaling—reduces caspase-3 activation and limits cytokine- or oxidative stress–induced apoptosis, highlighting how a favourable Bcl-2/Bax balance can shift β-cells toward survival under stress conditions [142].

### 5.3. Metabolic Reprogramming Under Nutrient and Oxidative Stress: Can β-Cells Apply Similar Strategies in Hypoxia?

The regulation of β-cell function in the face of chronic nutrient overload involves a complex and dynamic reprogramming of metabolic pathways that extends well beyond the activation of glycolysis and the tricarboxylic acid (TCA) cycle [143,144]. Recent evidence demonstrates that chronic exposure to hyperglycemic or hyperlipidemic conditions—as well as prolonged treatment with certain antidiabetic medications—induces various adaptations and maladaptations in pancreatic β-cells, including changes in the expression and activity of metabolic enzymes and transporters, as well as extensive epigenetic modifications [145,146]. These epigenetic changes encompass alterations in histone acetylation and methylation, DNA methylation patterns, and chromatin remodeling that collectively redefine the transcriptional landscape of β-cells in a manner that is tightly linked to their growth, survival, and insulin secretory capacity [145,146]. β-cell adaptive and protective mechanisms during nutrient-induced metabolic stress also involve post-translational modifications, notably O-GlcNAcylation; aberrant O-GlcNAc cycling is recognized as a nutrient and stress-sensitive regulator with both protective and pathophysiological roles in β-cells [147,148,149]. Increased O-GlcNAcylation, in particular, has been implicated in both adaptive and maladaptive responses to glucotoxicity, influencing signaling pathways that govern oxidative stress and cell survival [147]. Chronic glucotoxicity also leads to aberrant activation and mislocalization of small GTPases such as Rac1, resulting in mitochondrial dysfunction, increased generation of reactive oxygen species (ROS), and activation of stress kinases that can promote apoptosis [150]. In addition, the RhoG-Rac1-dependent signaling cascade illustrates how hyperglycemic stress can trigger metabolic reprogramming that predisposes β-cells to functional decline and death [151].

Moreover, nutrient-induced metabolic stress is closely linked to changes in mitochondrial dynamics and bioenergetics. Recent studies using live-cell imaging and computational modeling have highlighted that the balance between mitochondrial fission and fusion is critical for maintaining β-cell function, as these processes directly impact the efficiency of ATP production and the handling of metabolic substrates [152,153]. Additionally, regulators such as LKB1 serve as crucial molecular links by coupling glucose metabolism to insulin secretion; under altered nutrient availability, LKB1 deficiency can impair the inhibition of key lipogenic enzymes, thereby exacerbating metabolic stress [154].

Protective strategies involving natural compounds have also emerged as promising modulators of β-cell metabolic reprogramming in states of glucolipotoxicity. For example, treatment with extracts derived from *Siegesbeckia orientalis* has been shown to mitigate ROS accumulation, enhance intracellular antioxidant capacity, and stabilize the metabolic machinery of β-cells, counteracting the detrimental effects of high-glucose conditions [155]. Collectively, these advances underscore the multifaceted nature of metabolic reprogramming in pancreatic β-cells. Additional research is required to determine whether β-cells actively utilize extensive epigenetic and post-translational regulatory mechanisms crucial for adaptation to hypoxic stress. However, a previous study showed that hypoxia suppressed the adaptive UPR in β-cells via JNK and DDIT3 activation, impairing ER-to-Golgi trafficking and promoting apoptosis, independent of HIF1α. Overexpression of adaptive UPR gene *Hspa5* restored UPR function and significantly protected β-cells from hypoxia-induced death [156], suggesting a potential target to restore β-cell function in hypoxia.

Of note, most of the adaptive mechanisms taken on by β-cells under nutrient or oxidative stress overlap with those initiated during hypoxia. This significantly converges with hypoxia-inducible factor (HIF) signaling. HIF-1α functions as a central metabolic sensor by associating oxygen and nutrient status in order to ensure glycolytic flux and downregulate mitochondrial oxidative metabolism [28]. In environments of maternal metabolic stress or ongoing glucolipotoxicity, sustained HIF-1α stabilization has been shown to subdue β-cell transcriptional regulators such as *Pdx1*, *Mafa*, and *Nkx6.1*. This leads to a weakened mitochondrial function and reduced *Insulin* gene expression [157,158]. Moreover, HIF-1α interacts with chromatin-modifying enzymes and non-coding RNAs to initiate the coupling of redox and oxygen signals and ensure durable epigenetic reprogramming of β-cell identity and function [159]. The interaction between both metabolic and hypoxic stress suggests that β-cells may use regulatory frameworks that are similar. These include metabolic rewiring, transcriptional control, and epigenetic remodeling, which allow them to adapt to both nutrient excess and oxygen deprivation [160]. However, when these pathways are perpetually engaged, there is a tendency to shift from adaptive to maladaptive processes, thereby predisposing β-cells to impaired function and loss of plasticity in the future [6,28].

### 5.4. Mitophagy as an Adaptive Response to Hypoxic Stress in β-Cells

Hypoxic stress activates mitophagy in β-cells, often through the stabilization of hypoxia-inducible factor-1α (HIF-1α) and signals linked to increased mitochondrial ROS. This process prevents the buildup of dysfunctional mitochondria that could otherwise escalate cellular stress and β-cell death [161]. Mitophagy constitutes a crucial adaptive response in pancreatic β-cells under hypoxic stress by selectively removing damaged or dysfunctional mitochondria and thereby preserving cellular homeostasis and function [94,139,162]. Under conditions of low oxygen, HIF-1α is stabilized and activated, which initiates a transcriptional response that includes the upregulation of mitophagy mediators such as BNIP3 [163]. BNIP3 functions as a receptor on the outer mitochondrial membrane, directly linking hypoxic signaling to the autophagic machinery to target impaired mitochondria for degradation [164]. This receptor-mediated mitophagy is essential in β-cells because mitochondrial dysfunction is directly connected to reactive oxygen species accumulation and defective insulin secretion [95,139]. In addition to BNIP3-mediated pathways, studies have highlighted the role of mitophagy adaptors and regulators such as Parkin and Clec16a in pancreatic β-cells. The activation of CLEC16A specifically counteracts the apoptosis of β-cells induced by inflammatory cytokines, showcasing potential effects in preventing diabetes [139,165]. Recently, the Plk3-mtROS-PINK1-Parkin axis, as a new regulatory process of mitochondrial autophagy, has been shown to inhibit β-cells growth and insulin secretion, suggesting the significance of enhancing mitochondrial autophagy as a potential therapeutic strategy [166]. A recent study has demonstrated that, under hypoxic or metabolic stress, Ca^2+^ is released from lysosomes, activating the phosphatase calcineurin, which in turn stimulates the transcription factor EB (TFEB) [167]. TFEB is a master regulator of both autophagy and lysosomal biogenesis genes, promoting mitophagy and thereby maintaining mitochondrial quality [94]. Furthermore, the mechanistic target of rapamycin (mTOR) pathway, a central regulator of cellular metabolism, can be a mitophagy-modulator under hypoxic stress in β-cells. Inhibition of mTOR (e.g., by hypoxia or pharmacological agents like rapamycin) induces autophagy, including mitophagy, helping preserve β-cell mass and reduce apoptosis [39].

### 5.5. HIF-1α–Mediated Oxygen Utilization Strategies: Do β-Cells Employ Similar Mechanisms to Other Hypoxia-Adapted Cells?

**Figure 4:**
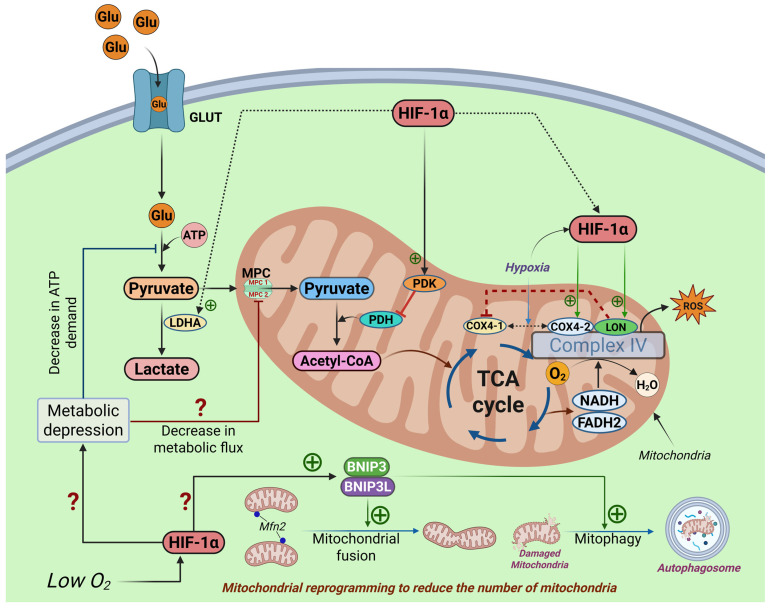
Under hypoxic conditions, pancreatic β-cells are proposed to undergo coordinated metabolic and mitochondrial reprogramming orchestrated by hypoxia-inducible factor 1α (HIF-1α). HIF-1α upregulates pyruvate dehydrogenase kinase (PDK), which phosphorylates and inhibits the pyruvate dehydrogenase (PDH) complex, thereby reducing acetyl-CoA supply to the tricarboxylic acid (TCA) cycle and limiting mitochondrial oxygen consumption. In parallel, lactate dehydrogenase A (LDHA) diverts pyruvate to lactate, regenerating NAD^+^ to sustain glycolytic ATP production when oxygen is scarce. HIF-1α also fine-tunes electron transport chain (ETC) activity by promoting a subunit switch in complex IV from COX4-1 to COX4-2 and inducing the mitochondrial protease LON to degrade COX4-1, which together optimize cytochrome c oxidase efficiency and restrain reactive oxygen species (ROS) generation. At the whole-cell level, β-cells may additionally enter metabolic depression, actively lowering ATP demand by reducing glycolytic flux and/or limiting pyruvate entry into mitochondria via the mitochondrial pyruvate carrier (MPC), thus decreasing oxygen requirements. Mitochondrial reprogramming under low O_2_ is further illustrated by HIF-1–dependent mitophagy and mitochondrial fusion: BNIP3 and BNIP3L (NIX) act as mitophagy receptors on damaged mitochondria, while mitofusin 1 (Mfn1) promotes mitochondrial fusion. The unanswered questions are whether β-cells exhibit metabolic depression (actively reducing ATP demand or by decreasing metabolic flux into mitochondria) or mitochondrial reprogramming (to reduce the number of mitochondria) to optimize oxygen utilization, preserve mitochondrial integrity, and maintain essential functions during hypoxia. Green ⊕ indicates activation. Glu, glucose; GLUT, glucose transporter; LDHA, lactate dehydrogenase A; MPC, mitochondrial pyruvate carrier (MPC1/MPC2); PDK, pyruvate dehydrogenase kinase; PDH, pyruvate dehydrogenase; COX4-1/COX4-2, cytochrome c oxidase subunit 4 isoforms 1 and 2; LON, Lon peptidase 1 (mitochondrial protease); BNIP3, BCL2/adenovirus E1B 19 kDa protein-interacting protein 3; BNIP3L, BNIP3-like protein (NIX); Mfn1, mitofusin 1; TCA, tricarboxylic acid; ROS, reactive oxygen species; NADH, reduced nicotinamide adenine dinucleotide; FADH2, reduced flavin adenine dinucleotide; ATP, adenosine triphosphate. A standard arrowhead, irrespective of its color, usually denotes stimulation or a positive effect. In contrast, a blunt or flat-ended line (┴) generally represents inhibition or a negative effect.

Under hypoxic conditions, the transcription factor hypoxia-inducible factor 1 (HIF-1) serves as a master regulator by targeting key metabolic enzymes, notably lactate dehydrogenase A (LDH-A) and pyruvate dehydrogenase kinase 1 (PDK1) [168,169]. PDK1 phosphorylates the catalytic subunit of the pyruvate dehydrogenase (PDH) complex, leading to its inactivation and inhibiting the conversion of pyruvate to acetyl-CoA. This reduction in PDH activity prevents pyruvate entry into mitochondria, thereby limiting acetyl-CoA production and suppressing tricarboxylic acid (TCA) cycle flux [170,171]. Simultaneously, elevated LDH-A expression promotes the conversion of pyruvate to lactate using NADH, thereby regenerating NAD+ to sustain a high glycolytic rate, which is particularly beneficial during oxygen scarcity [172] (Figure 4). In pancreatic β-cells, such a shift in pyruvate metabolism has critical implications for insulin secretion and overall cell function. Studies in β-cell models, such as MIN6 cells, have demonstrated that overexpression of LDH-A can influence glucose-induced insulin secretion, highlighting the delicate balance required in regulating glycolysis under hypoxic stress [173,174]. Furthermore, evidence from investigations on Von Hippel–Lindau (VHL) protein deficiency in β-cells supports that impaired glucose metabolism—partly due to dysregulated HIF-1 signaling that upregulates both PDK1 and LDH-A—can lead to β-cell dysfunction and disturbances in glucose homeostasis [175,176]. Therefore, the exact mechanism by which β-cells exhibit a distinct metabolic plasticity that enables them to maximize oxygen utilization during hypoxia is yet to be identified; however, the key mechanism should essentially include the selective downregulation of mitochondrial oxygen consumption. As demonstrated in general cellular models, HIF-1α can modulate ETC complex IV via COX4 subunit switching. Under normoxia, cells predominantly express COX4-1, whereas hypoxia triggers a switch to COX4-2. HIF-1 activates transcription of *COX4-2* and *LON*, a mitochondrial protease required for COX4-1 degradation, thereby optimizing cytochrome c oxidase activity and restraining ROS generation in hypoxic conditions (Figure 4) [177]. In pancreatic β-cells, which are metabolically active and heavily reliant on oxygen supply for insulin secretion and mitochondrial ATP production, such fine-tuning of the ETC is crucial for sustaining function during hypoxia [11]. The metabolic reprogramming in β-cells ensures that even when oxygen is scarce, the limited supply is used judiciously to support ATP-dependent processes, including insulin biosynthesis and secretion [17]. Moreover, pancreatic β-cells can reduce energy expenditure through ‘metabolic depression’, an adaptive mechanism that lowers overall ATP demand by down-regulating ATP-consuming pathways. This may occur either by slowing the basal glycolytic rate or by limiting pyruvate entry into mitochondria, thereby decreasing oxygen requirements (Figure 4). Such metabolic downregulation has been observed in other cell types and involves an active decrease in metabolic flux through the mitochondria, further contributing to the conservation of oxygen and reduction in harmful ROS leakage [178]. In addition, mitochondrial reprogramming under hypoxic conditions largely depends on HIF-1–mediated mitophagy across diverse cell types. This hypoxia-induced process is driven by HIF-1α regulation of BNIP3 translocation to mitochondria [179], where BNIP3 acts as a receptor-mediated mitophagy inducer, thereby reducing mitochondrial abundance and preventing excessive ROS accumulation (Figure 4). Pancreatic β-cells may similarly engage mitochondrial reprogramming to cope with altered metabolic pressure under hypoxia, although this possibility still requires direct experimental validation in β-cell models. Beyond mitophagy, HIF-1–driven mitochondrial reprogramming in cancer cells includes a shift toward mitochondrial fusion and enhanced survival: by targeting BNIP3 and BNIP3L at the mitochondrial membrane and upregulating Mfn1, these cells remodel mitochondrial dynamics, evade apoptosis, and gain a selective growth advantage. In parallel, the induction of glycolytic enzymes helps to partially compensate for the decreased flux through oxidative phosphorylation, ensuring that energy production maintains a level sufficient for cell survival and function during hypoxic periods [180]. Taken together, HIF-1α-mediated mitochondrial reprogramming, metabolic depression to reduce ATP demand, and compensatory upregulation of glycolysis should be explored in β-cells to elucidate the mechanism of maximum oxygen utilization in hypoxia. The adaptive and maladaptive responses of β-cells to hypoxia have been summarized in Table 1.

### 5.6. Molecular Regulators Supporting β-Cell Survival in Hypoxia

#### 5.6.1. Redox Regulation via Glutathione Peroxidase (GPx)

Hypoxia leads to mitochondrial dysfunction and elevated production of harmful ROS such as hydrogen peroxide. Pancreatic β-cells are inherently vulnerable to oxidative stress during hypoxia due to their low basal antioxidant capacity, making redox regulation by glutathione peroxidases (GPXs) critical for their survival and insulin secretory function. GPX detoxifies hydrogen peroxide (H_2_O_2_) by reducing it to water using reduced glutathione (GSH), thereby preventing oxidative modification of redox-sensitive proteins, preserving mitochondrial membrane potential, sustaining ATP production, and maintaining glucose-stimulated insulin secretion [181]. Furthermore, evidence from other cells (myoblast cells or mouse brain) under hypoxic conditions suggests that therapies or preconditioning strategies enhancing GPx activity can lead to improved cell viability and reduced protein oxidation [182,183]. The expression level of GPx4 in β-cells was distinctly higher than in other islet cell types, and the extraordinarily high abundance of GPx4 implies an essential role of this enzyme in protecting β-cells against oxidative injury by preventing lethal accumulation of membrane lipid peroxides [184]. GPX4, uniquely capable of reducing lipid hydroperoxides within membranes, protects β-cells from iron-dependent lipid peroxidation and ferroptosis, processes that are exacerbated under hypoxia when mitochondrial ROS generation and membrane oxidation are increased [184,185]. Enhanced expression or activity of GPx has been shown to preserve β-cell viability and function by maintaining the redox balance within these cells. For example, incretin hormones such as GLP-1 and its analogs have been shown to increase the expression of GPx in β-cells, thereby contributing to their cytoprotection and improved insulin secretion dynamics [186]. Thus, it is highly likely that the specialized upregulation of GPx may provide structural protection to the β-cell membrane and support the preservation of insulin secretory function in hypoxia, where oxidative challenges are elevated.

#### 5.6.2. Growth Factor Modulation via IGFBP1

Under hypoxic conditions, pancreatic β-cells activate a series of stress-response pathways that include modulation of growth factor signaling, with insulin-like growth factor binding protein 1 (IGFBP1) potentially playing a protective role. IGFBP1 controls the bioavailability of insulin-like growth factors (IGFs), particularly IGF-1, by binding to them and modulating their interaction with the IGF-1 receptor (IGF1R) [187]. Under hypoxia, the expression and phosphorylation state of IGFBP1 may shift, altering IGF signaling. In some systems, the phosphorylated form of IGFBP1 under hypoxia binds IGF-1 more tightly, reducing IGF-1’s activation of its receptor and slowing cell proliferation. However, this can be protective—favoring cell survival over proliferation in resource-limited, hypoxic conditions [188]. In other contexts, and with different phosphorylation patterns, IGFBP1 can release IGF-1 to promote survival via IGF1R-mediated anti-apoptotic signaling [189]. IGFBPs contribute to the survival of pancreatic cancer cells under severely hypoxic conditions [190]. This is partly regulated through hypoxia-inducible factors (HIFs), which bind to the IGFBP1 promoter and increase its transcription under low oxygen conditions [191]. The expression of IGFBP1 in β-cells can be influenced by transcription factors that are sensitive to metabolic and hypoxic stress. For instance, under stress conditions, FoxO1 becomes modified via O-GlcNAcylation, which enhances its transcriptional activity and drives the expression of the *Igfbp1* gene in β-cells [192]. IGFBP1 possesses an RGD (Arg-Gly-Asp) sequence that allows it to interact with integrins (notably α5β1). Engagement of these integrins activates focal adhesion kinase (FAK) and downstream survival pathways, independently of IGFs [189]. This integrin-mediated signaling can promote cytoskeletal stability and cellular adhesion, further protecting β-cells from stress-induced death [193]. In animal models, increased IGFBP1 levels have been shown to enhance β-cell regeneration and recovery, especially after injury or during metabolic stress. IGFBP1 promotes the formation of new beta cells within the islet, helping restore insulin-producing capacity and maintain glucose homeostasis [194]. Taken together, under hypoxic stress, increased IGFBP1 expression in pancreatic β-cells may modulate IGF signaling and activate integrin/FAK pathways, thus reducing apoptosis, stabilizing cell structure, and enhancing regenerative responses, contributing to improved β-cell survival and function.

#### 5.6.3. Protein Synthesis Control via DDIT4/REDD1

Regulated in development and DNA damage response 1 (REDD1) (also known as RTP801, DDIT4, and dig2) is a novel hypoxia-inducible factor (HIF)-1-responsive protein and a target gene of the p53 and p63 transcription factors [195,196]. It has been suggested that REDD1 may be a potential therapeutic target for metabolic disorders, including those related to pancreatic islet function [197]. The role of REDD1 has been reported in regulating the Akt/mTOR signaling pathway, which governs cell growth and metabolism in response to nutrients and growth factors [198]. Moreover, changes in mTORC1 signaling have been shown to affect REDD1 expression and protein stability in addition to affecting mTORC1 signaling [199]. Conversely, rapamycin treatment to inhibit mTORC1 decreased the half-life of REDD1, suggesting that REDD1 may play a role as a metabolic double agent depending on its duration of expression in different physiological and pathological contexts [198]. β-cells are uniquely susceptible to oxidative stress because they have low intrinsic antioxidant defenses. By suppressing energy-intensive and ROS-generating pathways, DDIT4/REDD1 is vital for maintaining β-cell integrity and insulin secretory function during and after hypoxic episodes, such as those encountered in diabetes or during islet transplantation [200,201].

#### 5.6.4. Cell Adhesion Mechanisms Supporting Hypoxia Tolerance

Several distinct categories of cell adhesion molecules, including cadherins and catenins, have been identified, initially in the cancer field, and the techniques or biomaterials that can enhance the regulation of those cell adhesion molecules are of great interest in islet transplantation. A method for culturing mouse and human islets in vitro on α5-laminins, the natural components of islet basement membranes, has been shown to enhance the efficacy of islet transplantation [202]. Other research suggested that incorporation of human amniotic epithelial cells (hAECs) into islet heterospheroids improves the secretory function and viability of islet cells both in conventional culture and in hypoxic conditions [203]. Another potential way to regulate islet adhesion is to use the rough, open-porous outer surface of the polysulfone capillary that provides a site well-suited for vascular tissue formation and may support prolonged islet function after transplantation [203]. That E-cadherin expression in malignant tumours was linked to metastasis and a poor prognosis stimulated research into the structure of this protein, as well as its relationships and regulatory elements [204]. However, knowledge about how to target cell adhesion mechanisms is scant [205]. The peptide growth factors, which exert a protective effect against the hypoxia prevalent in tumors and which, in turn, appear to promote genetic alterations within tumor cells, are one potential target. Combining a peptide growth factor inhibitor with an angiogenesis inhibitor may promote hypoxia in the target tumor, thereby preventing metastasis [206,207].

## 6. Endogenous and Exogenous Regulatory Factors Supporting β-Cell Survival Under Hypoxic Stress

### 6.1. GABAergic Neuronal Signaling: Potential for Enhancing β-Cell Function During Hypoxia

**Figure 5:**
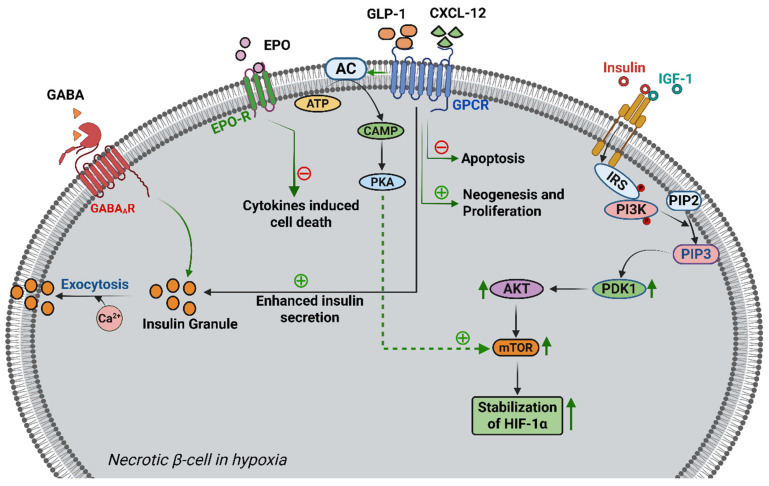
Schematic illustration of a necrotic β-cell and key factors promoting β-cell survival under hypoxic conditions. Human pancreatic β-cells express the GABA_A_ receptor (GABA_A_R), through which GABA enhances Ca^2+^-dependent insulin secretion. Erythropoietin (EPO) interacts with its receptor (EPO-R) expressed on β-cells, conferring protection against cytokine-induced cell death. Glucagon-like peptide-1 (GLP-1), in cooperation with C-X-C motif chemokine ligand 12 (CXCL-12), binds to their respective G-protein coupled receptors (GPCRs) on β-cells and exerts protective effects by reducing apoptosis, enhancing neogenesis and proliferation and boosting insulin secretion. Insulin and insulin-like growth factor-1 (IGF-1) bind to insulin receptors, activating insulin receptor substrate (IRS) and triggering phosphorylation of phosphoinositide 3-kinase (PI3K). PI3K converts phosphatidylinositol 4,5-bisphosphate (PIP2) to phosphatidylinositol 3,4,5-trisphosphate (PIP3), which in turn activates phosphoinositide-dependent kinase-1 (PDK1) and downstream AKT signaling. Activated AKT phosphorylates and activates mammalian target of rapamycin (mTOR). GLP-1 signaling can also stimulate mTOR via the cyclic adenosine monophosphate (cAMP)–protein kinase A (PKA) pathway. Under hypoxia, mTOR activation promotes the accumulation and stability of HIF-1α. AC, Adenylate cyclase. Green ⊕ indicates activation, red ⊖ indicates inhibition, upward green arrows denote activation of intracellular signaling molecules, and red ℗ marks phosphorylation events.

γ-Aminobutyric acid (GABA), which was initially identified as the major inhibitory neurotransmitter in the mammalian central nervous system (CNS), is also found in significant quantities in pancreatic β-cells [208,209]. GABA has been shown to play multiple roles in pancreatic β-cell function, such as modulating insulin secretion, protecting β-cells from oxidative stress and apoptosis, and promoting β-cell differentiation and proliferation [210,211,212,213]. The inhibitory function of GABA in the CNS is mainly mediated by two classes of GABA receptors, type A and type B (GABA_A_ and GABA_B_ receptors, respectively); however, in human islets, GABA_A_ receptor subunits were detected both in mRNA and protein levels [214]. In β-cells, the GABA_A_ R-mediated Ca^2+^-dependent PI3K/Akt pathway is the major mediator in exerting the excitatory action of insulin secretion [209]. A study performed in isolated rat islets subjected to intermittent hypoxia (IH) demonstrated GABA-enhanced insulin secretion [215] (Figure 5), suggesting GABA as an excellent insulin secretagogue and novel interventions for insulin regulation during IH of disordered breathing, including obstructive sleep apnoea (OSA).

### 6.2. β-Cell–Generated Serotonin: An Autocrine-Paracrine Modulator of Survival and Metabolic Adaptation in Hypoxia

Apart from GABA, pancreatic β-cells synthesize increased amounts of serotonin (5-hydroxytryptamine, 5-HT) through the expression of tryptophan hydroxylase (TPH1/TPH2), especially under conditions of metabolic or hypoxic stress [216,217]. Serotonin functions in an autocrine as well as a paracrine manner, thereby engaging certain 5-HT receptor groups (particularly 5-HT_2_B and 5-HT_3_) to govern β-cell proliferation, insulin secretion [218], and opposition to oxidative injury [219]. Throughout pregnancy and the early postnatal period, β-cell serotonin biosynthesis reinforces adaptive β-cell mass expansion and increases glucose-stimulated insulin secretion (GSIS) via serotonylation of exocytotic GTPases such as Rab3a and Rab27a [220]. In hypoxic conditions, HIF-1α activation can transcriptionally increase the expression of *TPH1*, thereby increasing intracellular serotonin levels and hence promoting β-cell survival by antioxidant and pro-survival signaling pathways [217,221]. However, ongoing or increased levels of serotonin signaling can disrupt β-cell maturation and lead to metabolic misprogramming. This thus emphasizes the need for finely balanced serotonergic tone during adaptation to hypoxia [221]. Thus, these findings indicate that β-cell–derived serotonin behaves as a locally generated factor that combines oxygen sensing, metabolic stress, and insulin secretory capacity, creating a potential therapeutic axis for conserving β-cell identity and function under hypoxic stress [217].

### 6.3. Erythropoietin-Mediated Cytoprotection in Hypoxic β-Cells

Erythropoietin (EPO), an important glycoprotein hormone, is produced by interstitial fibroblasts in the kidney in close association with peritubular capillary and tubular epithelial cells [222] and is necessary for growth, survival and differentiation. EPO binds to its corresponding receptor (EPO-R), which is mainly expressed on erythroid cells; however, non-erythroid tissues, including rodent and human pancreatic islets, also express EPO-R [223]. EPO overexpression in human pancreatic islets has been shown to protect against cytokine-induced cell death [224]. An increase in hypoglycemic events in clinical trials of EPO treatment in non-diabetic renal failure patients demonstrated a direct effect of EPO on pancreatic β-cells [223,224]. EPO treatment increased the functional capillary density and revascularization area of islet transplantation grafts in mice [225], suggesting EPO as a potent therapeutic agent in overcoming hypoxic damage to β-cells.

### 6.4. The Islet Microvascular Bed: Endothelial–Pericyte Interplay in β-Cell Survival

The islet microvascular bed is essential for maintaining β-cell function and survival, particularly under metabolic and hypoxic stress. It comprises intra-islet endothelial cells that not only deliver oxygen and nutrients but also act as paracrine regulators, releasing nitric oxide (NO), prostacyclin, connective tissue growth factor (CTGF), and thrombospondin-1 (TSP-1), which promote insulin secretion and protect β-cells from stress-induced damage [226]. Contractile pericytes surrounding the capillaries regulate local capillary blood flow and exhibit mesenchymal stem-cell-like properties, secreting trophic factors (basement-membrane components such as nerve growth factor (NGF), bone morphogenetic protein-4 (BMP4), and laminin-421 to sustain β-cell gene expression (*Ins1*, *MafA*, *Glut2*) and prevent de-differentiation upon their loss [227]. Thus, coordinated signalling among endothelial cells, pericytes, and macrophages establishes a regenerative niche that collectively ensures nutrient delivery, paracrine communication, and recovery following the injury. Disruption of this coupling represents an early hallmark of diabetic microvascular disease and contributes to β-cell dysfunction and loss [228]. Together, targeting this vascular-derived oxygen delivery and pericyte trophic signaling may therefore mitigate hypoxia-induced β-cell dysfunction, but often remains an underexplored determinant of β-cell resilience under hypoxic stress.

### 6.5. Incretin Hormones and Cytokines: Augmenting β-Cell Activity and Survival in Hypoxia

In patients with T2D, β-cell function gradually deteriorates. By the time of diagnosis, there may be a loss of up to 60% of β-cell mass, due to β-cell apoptosis, and a reduction in islet function of up to 50% [229,230]. Few pharmaceutical treatments now exist to address this decline in β-cell mass and function. After a meal, the digestive system releases incretin hormones (for example, glucagon-like peptide-1 (GLP-1) and glucose-dependent insulinotropic polypeptide (GIP)) that increase glucose-dependent insulin secretion, helping to regulate glucose homeostasis in healthy individuals. These hormones also have a number of additional protective effects on the β-cells, including a decrease in apoptosis and an increase in neogenesis and proliferation [231]. Exenatide and liraglutide, GLP-1 receptor agonists, are two recently developed diabetic treatments that show promise for long-lasting glucose control as well as for reducing the risk of micro- and macrovascular complications related to T2D [232]. Delivery of Exendin-4 (Ex-4) increased the rate of insulin secretion in normoxic and hypoxic conditions, as well as decreased apoptosis in hypoxic conditions within native pancreatic islets by acting in a paracrine manner. Furthermore, compared to Ex-4 alone, delivery of C-X-C motif chemokine ligand 12 (CXCL12) together with Ex-4 markedly boosted insulin secretion in hypoxic conditions (Figure 5). These data show that a promising strategy for promoting β-cell activity and survival after transplantation may involve treatment with CXCL12 in conjunction with Ex-4 [233,234,235].

### 6.6. cAMP–mTOR Signaling and HIF-1α Stabilization in β-Cells Under Hypoxic Stress

The stability and activity of HIF-1α have important implications for an array of biological functions, especially angiogenesis, cell survival and metabolism. mTOR signaling helps HIF-1α stability through various mechanisms. Previous research suggests that the prolyl hydroxylase (PHD)-HIF feedback loop limits the induction of HIF-1α by mTOR [236]. mTOR signaling also plays a role in stabilizing HIF-1α in pancreatic β-cells. It has been reported that GLP-1 triggers the cAMP pathway in β-cells, which activates mTOR and promotes the accumulation of HIF-1α [237]. It has been suggested that modulating mitochondrial oxidative stress and ER stress via mTOR signaling may be an approach to alleviate metabolic disorders such as T2D [238]. Another study found that sumoylation increases HIF-1α stability and its transcriptional activity [239]. Normoxic HIF-1α activity can be upregulated through NO-mediated S-nitrosylation and stabilization of HIF-1α [240]. Intracellular cAMP also promotes HIF-1α stability in pancreatic β-cells through various mechanisms. cAMP promotes pancreatic β-cell survival via CREB-mediated induction of IRS2, which enhances activation of the survival kinase Akt in response to insulin and IGF-1 [241]. cAMP triggers a second delayed phase of gene expression that proceeds via the HIF-1α transcription factor and increases in cAMP promote the accumulation of HIF-1α in β-cells by activating the mTOR pathway [237] (Figure 5). These findings suggest that cAMP promotes HIF-1α stability in pancreatic β-cells through the mTOR pathway. HNF transcription factors, including HNF-1α, play a role in regulating gene expression in pancreatic islets and may also contribute to the regulation of HIF-1α stability [242].

### 6.7. Pharmacological Inhibition of ChREBP to Enhance ARNT/HIF-1β Activity in Hypoxic β-Cells

**Figure 6:**
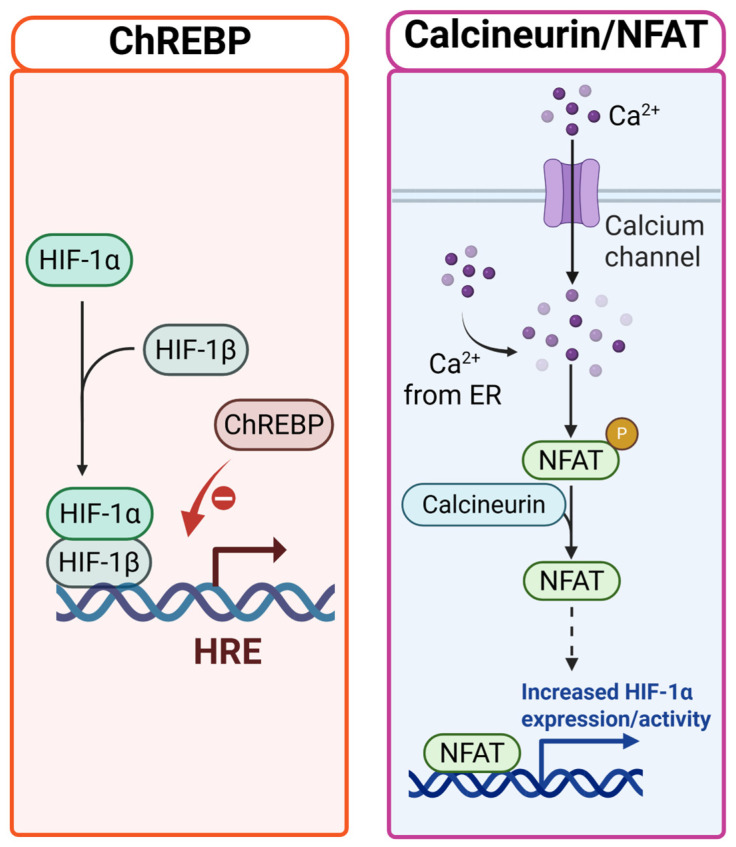
Regulation of hypoxia-inducible factor-1α (HIF-1α) transcriptional activity by carbohydrate-responsive element-binding protein (ChREBP) and the Ca^2+^-dependent calcineurin/nuclear factor of activated T cells (NFAT) pathway in pancreatic β-cells. **Left** panel (ChREBP): HIF-1α dimerizes with HIF-1β (aryl hydrocarbon receptor nuclear translocator, ARNT) and binds hypoxia-response elements (HREs) to drive target gene transcription. ChREBP can associate with this complex and act as a negative regulator, limiting HIF-1–dependent transcription. Inhibition or downregulation of ChREBP relieves this repression, thereby enhancing ARNT/HIF-1β expression and promoting HIF-1α/HIF-1β transcriptional activity. **Right** panel (Calcineurin/NFAT): Hypoxic or depolarizing stimuli increase intracellular Ca^2+^ through plasma membrane calcium channels and release from the endoplasmic reticulum (ER). Elevated Ca^2+^ activates calcineurin, a Ca^2+^/calmodulin-dependent phosphatase, which dephosphorylates NFAT. Dephosphorylated NFAT translocates to the nucleus, where it binds DNA and upregulates genes that increase HIF-1α expression and/or activity, thereby amplifying the hypoxic transcriptional response.

One of the key factors that regulates β-cell survival under hypoxic conditions is the transcription factor ARNT/HIF-1β, which activates genes involved in glucose metabolism and angiogenesis [243]. However, HIF-1β expression and activity are often reduced in diabetic β-cells due to the high prevailing glucose levels. One way to enhance HIF-1β expression and activity is to inhibit its negative regulator, ChREBP, which is a glucose-responsive transcription factor that suppresses *HIF-1β* gene expression [244]. High glucose also activates ChREBP-mediated HIF-1α and VEGF expression in human retinal pigment epithelial (RPE) cells under normoxia, which may be partially responsible for neovascularization in both diabetic and age-related retinopathy [245]. Therefore, pharmacological or transcription level (by RNA interference) inhibition of ChREBP could be beneficial for β-cells via increasing HIF-1β levels (Figure 6), thus protecting β-cells from hypoxic stress [246].

### 6.8. Calcineurin–NFAT Signaling: Can It Be Harnessed to Regulate HIF-1α in Hypoxic β-Cells?

The calcineurin-NFAT signaling pathway is a key regulator of cellular responses to stimuli such as calcium influx, hypoxia and cytokines. Calcineurin is a calcium-dependent phosphatase that dephosphorylates and activates nuclear factor of activated T cells (NFAT), a family of transcription factors that control gene expression in diverse cell types [247]. In pancreatic β-cells, the calcineurin-NFAT pathway has been implicated in glucose-induced insulin secretion, β-cell survival and adaptive β-cell mass expansion [248,249]. However, the role of this pathway in hypoxic adaptation of β-cells is not well understood. Recent studies have suggested that the calcineurin-NFAT pathway can modulate HIF-1α expression and activity in various cell types, such as cardiomyocytes, endothelial cells and intervertebral disc cells [250] (Figure 6). Therefore, targeting the calcineurin-NFAT pathway to regulate HIF-1α in pancreatic β-cells might have potential implications for β-cell function and survival under hypoxic stress.

### 6.9. Neuroglobin and Cytoglobin Activation: Potential Oxygen-Handling Mechanisms in Hypoxic β-Cells

Neuroglobin (Ngb) and Cytoglobin (Cygb) are two globin proteins that are expressed in various tissues, including islets or β-cells [251]. Hypoxia-tolerant species express more neuroglobin in their brains than their more oxygen-deprivation sensitive relatives [252]. Moreover, sustained hypoxia increased neuroglobin mRNA and protein expression throughout exposure [253]. Cytoglobin is also upregulated upon exposure to hypoxia in all tissues [254]. Ngb and Cygb have been proposed as protective mechanisms against hypoxic stress in islets or β-cells [255,256]. They may function by enhancing oxygen delivery, scavenging ROS, modulating gene expression, or preventing apoptosis [257]. It has been suggested that neuroglobin may play a myoglobin-like role, supplying oxygen to the respiratory chain of metabolically active neurons or protecting them from ROS. Cytoglobin, on the other hand, is involved in cell proliferation and collagen synthesis in fibroblasts and has a role related to nitric oxide metabolism in neurons [252]. Therefore, its administration or upregulation would likely improve β-cell survival and function under hypoxic conditions.

## 7. Strategies to Mitigate Hypoxia in Pancreatic β-Cell and Islet Transplantation

Adequate oxygen is critical for the survival and healthy functioning of pancreatic islets, the lack of which negatively impacts GSIS. To ensure adequate oxygen delivery, islets in the native pancreas are well perfused with a dense capillary network, assuring abundant oxygen delivery via blood circulation to all islet cells. The oxygen tension surrounding islets in the pancreas is 30–40 mmHg and, because of the dense vascularity, can increase close to that of arterial blood (80–100 mmHg) [258]. The pancreatic islet is a cluster of, on average, 2000 cells, with highly specific requirements [259]. Key variables, such as islet size, local oxygen partial pressure (pO_2_), islet oxygen consumption, islet size (diameter, D), and the presence or absence of thrombosis on the islet surface, may have a profound impact on oxygen supply to the overall islet. Isolated human islets are not uniform in size and generally range from 50–500 μm in diameter (average 150 μm diameter) [260]. Average size distribution data from clinical alloislet preparations indicate that >150 µm diameter islets account for only ~30% of the total islet number but >85% of the total islet volume. This means that most of the β-cell volume is contained within large islets that are most susceptible to hypoxic conditions [261]. In large islets, oxygen tension is markedly lower (hypoxic) in the islet core versus the islet surface. Hence, the oxygen gradient-induced hypoxic core is enlarged in larger islets, contributing to the development of central necrosis in human isolated islets [260]. As β-cells secrete insulin in response to high glucose, the oxygen consumption rate increases. This microenvironmental change worsens the internal hypoxia in the islet. Healthy islets can rapidly restore normoxia without damage; however, in diabetic islets or islets post-transplantation, the microenvironment is disturbed, and hypoxia can induce necrosis [262].

### 7.1. Pre-Transplantation Hypoxia and Hyperoxia: Balancing Oxygen Supply for Islet Viability

Islets encounter hypoxia and oxidative stress due to the islet isolation procedure itself, as islets become disconnected from their blood supply. Not only does this impact insulin secretion, but it also induces inflammation and can ultimately cause cell apoptosis. β-cells are known to be sensitive to oxidative stress; therefore, viable islet preparations must be optimized with cytoprotective agents to reduce stress-induced cell death in the pre-transplant culture condition [263]. Conversely, hyperoxia (270–350 mmHg pO_2_) induces cell damage on the islet surface. Hyperoxia has been found to alleviate central necrosis when carefully applied to an islet culture. Recent studies have demonstrated that hyperoxic cultures (35% and 50% O_2_) maintain islet cell viability and function, though O_2_ toxicity occurs above 75% O_2_ (>500 mmHg pO_2_) [264].

### 7.2. Oxygenation Strategies for Pancreatic Islets to Improve Transplant Outcomes

To date, clinical islet transplantation has proven successful with insulin independence up to 5 years post-transplant with minimal complications [265]. However, oxygenation remains a hurdle, lack of oxygen contributing to poor islet yield as well as inducing cellular death/dysfunction pre-and post-transplantation. Interventions at each step of the transplantation process ensure adequate oxygenation and improve clinical islet transplantation outcomes.

Oxygenation can be achieved through many carriers, oxygen-generating materials, and factors that increase revascularization. Common oxygen carriers are semi-fluorinated alkanes, perfluoro decalin, and hemoglobin [266]. Recently, macroencapsulation devices that can generate oxygen and restore normoglycemia to hypoxic islets have been developed. One popular enriched medium macroencapsulation device, “OxySite”, increases localized oxygenation at the transplantation site upon contact with water, indicating its utility in maintaining islet oxygenation during the transplant revascularization phase [267,268]. Other macroencapsulation devices incorporate immobilized *Synechococcus lividus* (active thermophilic cyanobacteria) that produce oxygen by photosynthesis when illuminated by a light source integrated into the device [269]. Post-transplantation β-cell death can be limited by stimulating revascularization using angiogenic factors or bone-marrow-derived cells, such as mesenchymal stem cells and hematopoietic stem cells [266].

### 7.3. Ischemic (Hypoxia) Preconditioning: Enhancing β-Cell Functional Capacity Before Transplantation

Ischemic preconditioning (IPC), a technique that involves applying brief episodes of nonlethal ischemia and reperfusion to protect against a sustained episode of lethal ischemia, has been validated using animal models of coronary heart disease [270,271]. The cardioprotective mechanisms of IPC are based upon the activation of several signaling pathways that converge on a few mitochondrial targets, leading to altered cell metabolism and the inhibition of apoptosis [272]. A study performed in rats demonstrated that IPC was associated with improved islet cell recovery after cold ischemia [273]. Intermittent exposure to hypoxia stimulated insulin synthesis and secretion by pancreatic β-cells and activated de novo formation of these cells in the acinar tissue of both intact rats and rats with streptozotocin-induced diabetes, as well as inhibiting the destruction of β-cells in the latter animals [274]. The mechanism by which intermittent hypoxia (IH) stimulates pancreatic β-cell function may involve the induction of *IL-6* gene expression. Following IL-6 stimulation, β-cells overexpress regenerating (Reg) family genes as well as the hepatocyte growth factor (HGF) gene. Reg family proteins stimulate β-cell proliferation and HGF inhibits apoptosis of β-cells. As a result, β cell numbers are increased by IH [275]. In addition, hypoxia induction had a positive effect on islet endothelial cell (EC) morphology and survival with limited impact on β-cell metabolism, function and survival [123]. Taken together, hypoxia preconditioning can be an effective way to improve β-cell function and survival under severe hypoxia, such as occurs during islet transplantation.

### 7.4. Pharmacological Modulation of HIF-1α and Pro-Angiogenic Pathways

Pharmacological modulation of the HIF-1α pathway represents a mechanistically distinct strategy that enhances endogenous adaptive responses to hypoxia rather than supplying exogenous oxygen. The GLP-1 receptor agonist liraglutide has been shown to enhance HIF-1α expression in pancreatic islets, resulting in increased VEGF production and accelerated revascularization, with studies demonstrating approximately 70% increase in neovessel density at day seven post-transplant [125]. However, the therapeutic application of HIF pathway modulators requires careful titration, as excessive or prolonged HIF-1α activation can paradoxically impair β-cell function by shifting cellular metabolism excessively toward glycolysis and reducing glucose-stimulated insulin secretion [23,175,176]. The clinical translation of this approach is facilitated by the availability of already-approved medications such as liraglutide and semaglutide, which have well-established safety profiles in diabetes management, though specific prospective trials examining their impact on islet transplant outcomes remain to be conducted.

### 7.5. Oxygen Transporter Technologies for Delivering Oxygen to Hypoxic Graft Sites

Post-islet transplantation, a robust oxygen supply is essential to prevent cell death, especially during the critical time that the graft is being re-vascularized [276,277]. To accomplish this, an implantable oxygen transporter that uses diffusion potential to carry oxygen from the surrounding air to the hypoxic graft site has been devised [278]. The device consists of tubing that opens outside the body, coupled to a biocompatible silicone receptacle to contain the islets. In computer simulations and in vitro testing, the oxygen transporter raised the oxygen level inside grafts to >120 mmHg. In vivo testing in diabetic rats demonstrated that, when implanted subcutaneously, the oxygen transporter increased islet graft viability and function, and this may have clinical application [260,279].

### 7.6. Extracellular Matrix Enhancement Strategies

Extracellular matrix enhancement strategies address hypoxia tolerance through biomechanical signaling and metabolic reprogramming rather than direct oxygen provision. Human islets cultured on laminin-511–coated surfaces under hypoxic conditions show upregulation of glycolytic genes, maintenance of insulin secretory capacity, and reduced apoptosis, with TUNEL-positive cells decreased by approximately 60% compared to controls cultured without ECM supplementation [202,280]. The incorporation of decorin into islet transplant scaffolds provides additional benefits through modulation of transforming growth factor-β signaling, resulting in reduced peri-graft fibrosis thickness by 40–50% and improved long-term graft function in preclinical models (19476204, 10556766). Several bioengineered scaffolds incorporating laminin and other basement membrane components have entered Phase I safety trials, with preliminary data suggesting feasibility and safety through twelve-month follow-up periods [281].

### 7.7. Mesenchymal Stem Cell–Derived Components and Exosomes: Protecting Islets from Hypoxia During Transplantation

Drawbacks of MSC-based therapies include poor cell survival, restricted differentiation and dedifferentiation of cells, issues that restrict the practical use of MSC therapy. Therefore, the concept of cell-free therapy is appealing. Paracrine secretion of growth factors, cytokines, extracellular matrix proteins, and microRNA-containing exosomes are beneficial factors secreted by MSCs [282]. When neonatal porcine islet cell clusters (NICCs) subjected to hypoxia were treated with MSC media containing exosomes, an increase in live cells with a decrease in apoptosis-associated protein expression resulted [283]. Moreover, there was a drop in the ratio of LC3 I/II and phosphorylated AMPK, indicating reduced autophagy. The authors concluded that conditioned medium containing exosomes is superior to conditioned medium devoid of exosomes in protecting NICC viability and function under hypoxic conditions, suggesting the potential of MSC-derived exosomes for enhancing porcine islet survival and function in clinical islet xenotransplantation [284].

Exosomes produced from MSCs may be an effective means of delivering nutrients to grafted islets and may be developed into a method for enhancing islet viability and function post-transplantation [285]. Hypoxia preconditioning raises the concentration of MSC trophic factors in the conditioned medium; when islets are exposed to hypoxia-preconditioned medium in vitro, islet function was enhanced and apoptosis due to, for example, pro-inflammatory cytokines, was reduced. Therefore, several preconditioning methods may be useful for optimizing the exosome protocol.

### 7.8. Islet Encapsulation Technologies to Protect Against Hypoxia and Immune Attack

Cell encapsulation is a viable strategy that relies on the placement of endocrine cells within semi-permeable hydrogel matrices to protect them from immune attack and avoid the need for ongoing immunosuppression. Numerous islet encapsulation methods are currently available. The most common approach is using polymeric hydrogel microcapsules to shield islets from immune cell interaction. The pore size of the biomaterial membrane must allow small nutrients in and insulin out but prevent entry of immune cells and immunoglobulins. One innovation is amphiphilic bilirubin nanoparticles (BRNPs), wherein a hydrophobic molecule of bilirubin is attached to a hydrophilic polyethylene glycol (PEG); the PEG-bilirubin conjugates self-assemble into BRNPs that prevent chemically induced oxidative stress by scavenging ROS molecules and prevent recruited macrophages from damaging islet cells by inhibiting cytokine production [286]. In vivo experiments indicate that BRNP administration can markedly increase islet transplant survival when compared to bilirubin treatment alone. Another approach utilizing heparin-infused, ultrathin starPEG nanofilm (Hep-PEG), that binds covalently to the amine groups of the islet surface membrane, has been shown to enhance islet viability and to preserve insulin secretory function; in addition, Hep-PEG islet coating decreased the inflammatory response and enhanced the survival of islets exposed to pro-inflammatory cytokines [287].

### 7.9. Combination and Multi-Modal Strategies

Comparative analysis across these different approaches reveals that combination strategies addressing multiple facets of transplant hypoxia yield superior outcomes to single-modality interventions. Studies combining oxygen-generating biomaterials with ECM enhancement and MSC support have demonstrated graft survival rates exceeding 80% at 30 days post-transplant and restoration of normoglycemia using only 7000–9000 islet equivalents per kilogram compared to the 11,000–15,000 islet equivalents per kilogram typically required with standard transplantation protocols [288,289]. This 30–45% reduction in islet mass requirements represents a clinically significant advance that could substantially increase the number of patients who could benefit from each donor pancreas. The field is increasingly moving toward personalized combination approaches that integrate immediate oxygen supplementation during the critical avascular phase, pharmacological or cellular strategies to accelerate host revascularization, and biomaterial or ECM-based methods to enhance intrinsic β-cell hypoxia tolerance, with the ultimate goal of optimizing both short-term graft survival and long-term functional outcomes in clinical islet transplantation [290,291]. A summary of strategies to mitigate hypoxia in islet transplantation is captured in Table 2.

### 7.10. Limitations of Current Experimental Models and Translation Challenges

The majority of evidence regarding β-cell hypoxia tolerance and therapeutic interventions derives from rodent studies, in vitro human islet cultures, and small animal transplantation models, each with significant limitations that complicate clinical translation.

Mouse and rat islets differ substantially from human islets in size, with rodent islets measuring 50–150 μm in diameter compared to human islets at 100–400 μm [296]. Architecture also varies significantly, with rodent islets displaying a β-cell core surrounded by α-cells while human islets have intermixed cell types [297]. Vascularity differs dramatically, with human islets having five-fold fewer vessels per islet area than mouse islets, and rodent β-cells have higher proliferative capacity [298]. These differences profoundly impact hypoxia tolerance because a larger human islet diameter creates greater oxygen diffusion distances, increasing susceptibility to core necrosis [299], architectural differences may alter paracrine signaling during stress, and lower human β-cell proliferative capacity limits regenerative responses post-injury. Consequently, interventions effective in rodent models may not translate to human islets.

Further, experimental islet transplantation studies employ diverse approaches, including intrahepatic versus subcutaneous versus omental sites, different islet numbers, varying degrees of purification, and inconsistent cold ischemia times that confound cross-study comparisons [300,301]. The intrahepatic site commonly used in clinical transplantation is physiologically distinct from subcutaneous sites used in many rodent experiments, particularly regarding oxygen tension with hepatic portal vein pO_2_ around 50 mmHg versus subcutaneous tissue at 25–35 mmHg [302], inflammatory milieu, including instant blood-mediated inflammatory reaction in liver, and immunological environment. This discordance limits the predictive value of preclinical efficacy data.

While oxygen-generating biomaterials and encapsulation devices show promise in controlled laboratory settings, real-world implementation faces multiple hurdles. Scalability presents a challenge because manufacturing GMP-grade biomaterials and devices at clinically relevant scales supporting at least 300,000 islet equivalents for adult patients while maintaining quality control is technically demanding and costly [268,303]. Reproducibility is affected by batch-to-batch variability in polymer properties, oxygen-release kinetics, and biocompatibility that affects device performance [304]. Regulatory approval requires novel combination products comprising cells plus biomaterials to undergo extensive preclinical testing, including biocompatibility according to ISO 10993 standards [305], sterility, dose-ranging studies, and demonstration of safety and efficacy in large animal models before clinical trials, adding years to development timelines. Clinical practicality issues arise because device implantation may require surgical procedures, increasing patient risk and cost, device retrieval or replacement is invasive, and long-term monitoring for device failure or fibrotic encapsulation demands imaging modalities not yet standardized [306,307].

Moving on, MSC-derived exosome preparations lack standardization across tissue source, donor characteristics, culture conditions, and isolation methods, leading to poorly reproducible experimental outcomes [308]. The field requires consensus on minimal criteria for MSC characterization, including immunophenotype, differentiation potential, and secretome profile; standardized exosome isolation and characterization protocols covering protein content, RNA cargo, and particle size distribution; validated potency assays correlating exosome bioactivity with therapeutic outcomes; and establishment of reference materials for quality control. Without this standardization, clinical translation remains premature.

Most preclinical studies report short-term outcomes lasting days to weeks due to cost and logistical constraints, whereas clinical islet transplantation aims for multi-year graft survival [300]. Interventions that improve early graft survival may have unforeseen long-term consequences, including chronic inflammation from biomaterial degradation, progressive fibrosis, or metabolic exhaustion of persistently hypoxic β-cells. Only longitudinal studies in large animals, particularly non-human primates, with extended follow-up of at least one year, can adequately assess the durability of therapeutic effects, yet such studies are rarely conducted due to expense and ethical considerations.

To bridge the translational gap, the field should prioritize development of humanized mouse models with transplanted human islets under clinically relevant conditions, establishment of multicenter consortia to standardize experimental protocols and enable cross-validation, increased use of non-human primate models for pivotal preclinical studies, early engagement with regulatory agencies to define appropriate endpoints and trial designs, and transparent reporting of negative and neutral results to prevent publication bias and guide future research directions.

## 8. Conclusions and Future Perspectives

Hypoxia represents a critical determinant of pancreatic β-cell fate, influencing insulin biosynthesis, secretion, metabolic coupling, mitochondrial function, and survival. While β-cells possess inherent adaptive mechanisms—such as HIF-mediated glycolytic reprogramming, autophagy, mitophagy, and antioxidant defense—these responses are often insufficient under chronic or severe oxygen deprivation, as seen in T2D, islet amyloidosis, OSA, and during islet transplantation. The intricate interplay between oxygen sensing, redox regulation, mitochondrial bioenergetics, and growth factor signaling underscores the complexity of β-cell adaptation to hypoxia. Emerging strategies—including oxygen-generating biomaterials, macroencapsulation devices, ischemic preconditioning, growth factor supplementation, and modulation of hypoxia-response pathways—have demonstrated promise in preclinical and translational studies. However, their long-term efficacy, safety, and integration into clinical protocols remain key challenges.

Looking ahead, multi-omics approaches, including transcriptomics, proteomics, metabolomics, epigenomics, and single-cell multi-omics, offer unprecedented opportunities to dissect the hypoxia-induced molecular signatures in β-cells with spatial and temporal resolution. By integrating these datasets, researchers can identify master regulators of hypoxia adaptation, novel biomarkers of β-cell stress, and therapeutic targets to enhance oxygen resilience. Spatial transcriptomics combined with multiplex imaging could further clarify oxygen gradient–dependent changes within intact islets, enabling targeted microenvironmental interventions. The incorporation of artificial intelligence (AI) and machine learning (ML) into β-cell hypoxia research will be transformative. AI-driven predictive modeling can integrate omics datasets, live-cell imaging outputs, and clinical parameters to forecast β-cell survival trajectories under varying hypoxic conditions. Deep learning algorithms applied to high-content microscopy or metabolic flux imaging can automate the detection of early hypoxia-induced dysfunction, accelerating drug screening for oxygen-sensitizing compounds. Furthermore, AI-assisted computational fluid dynamics models can optimize oxygen delivery strategies in islet transplantation devices, improving graft survival rates.

On the technological front, cutting-edge research tools such as CRISPR/Cas9-based gene editing, optogenetics for metabolic control, biosensors for real-time oxygen and ATP monitoring, and microfluidic “islet-on-chip” systems will refine our understanding of hypoxia-induced β-cell pathophysiology. These platforms enable precise manipulation of oxygen tension and nutrient availability, closely mimicking in vivo conditions, and providing high-throughput capacity for therapeutic testing.

Ultimately, the integration of systems biology, omics-driven discovery, AI-powered analytics, and advanced bioengineering holds the potential to not only elucidate the mechanisms underlying β-cell adaptation to hypoxia but also to translate these insights into next-generation therapies. Such an interdisciplinary approach could pave the way toward improving β-cell resilience, optimizing islet transplantation outcomes, and mitigating the progression of diabetes in hypoxia-compromised microenvironments.

## Figures and Tables

**Table 1:** Adaptive and Maladaptive Hypoxia-Response Pathways in Pancreatic β-Cells: Mechanisms, Biomarkers, and Translational Targets.

Response (Short)	Mechanism/Markers	Short-Term Effect (Adaptive)	Long-Term Effect (Maladaptive)	Readout/Markers to Measure	Translational Levers (Examples)
HIF-1α glycolytic switch	↑HIF-1α → ↑PDK1, ↑LDH-A, ↑GLUT1	Maintains ATP when O_2_ low; supports survival.	Chronic PDH inhibition → ↓TCA flux, loss of GSIS, lactate accumulation.	HIF-1α, PDK1, LDH-A mRNA/protein; lactate; PDH activity	Temporally tuned HIF stabilizers (early graft), PDK inhibitors (if chronic)
Mitophagy/BNIP3/PINK1-Parkin	BNIP3, PINK1 stabilization; TFEB activation	Removes damaged mitochondria → lowers mtROS, preserves ATP coupling.	Excessive mitophagy → loss of mitochondrial mass, impaired bioenergetics.	LC3 flux, BNIP3, PINK1, Parkin, mitophagy reporters (mtKeima)	TFEB activators, mitophagy modulators; careful dosing to avoid over-clearance.
Mitochondrial dynamics (fission/fusion)	↑DRP1-mediated fission under stress	Isolates damaged mitochondria for removal.	Excessive fission → fragmented network, reduced ATP, apoptosis.	DRP1 phosphorylation, MFN1/2 levels, mitochondrial morphology	DRP1 inhibitors (short window), promote fusion (MFN agonists)
Redox buffering (GPx, glutathione)	↑antioxidant enzymes; GPX4 protects membranes	Limits lipid peroxidation and ferroptosis; preserves integrity.	If insufficient → overwhelming ROS → protein/mtDNA damage, apoptosis.	GSH:GSSG ratio, GPx activity, lipid peroxides (MDA), 4-HNE	Boost antioxidants (GLP-1 effectors, GPx mimetics), ferroptosis inhibitors.
ER/UPR response	Adaptive UPR (HSPA5/BiP) vs. DDIT3/CHOP activation	Restores proteostasis, supports proinsulin processing.	Persistent UPR → DDIT3/CHOP → impaired ER-to-Golgi trafficking, apoptosis.	HSPA5, CHOP/DDIT3, XBP1 splicing, proinsulin:insulin ratio	Enhance adaptive UPR (HSPA5 overexpression in models), reduce ER stress (chemical chaperones).
Metabolic depression/energy saving	Downregulate ATP-consuming processes, adjust ion pumps	Lowers O_2_ demand, buys time for revascularization.	Chronic depression → loss of secretory responsiveness (reduced GSIS).	ATP/ADP ratio, KATP activity, gene signature of metabolic downregulation	Temporary metabolic depression strategies for transplant window; reversible modulators.
Angiogenesis (VEGF via HIF)	HIF → VEGF, Notch signaling → neovascularization	Restores perfusion and oxygenation (good for grafts).	Dysregulated angiogenesis → leaky vessels, fibrosis, maladaptive remodeling.	VEGF expression, capillary density, vessel maturity markers	Controlled pro-angiogenic therapy (timed VEGF delivery, MSC paracrine support).
Neuroglobin/Cytoglobin upregulation	↑Ngb/Cygb expression (oxygen binding/scavenging)	Improved O_2_ handling, ROS scavenging in hypoxia	Unknown long-term; potential metabolic tradeoffs	Ngb/Cygb expression; oxygen consumption assays	Consider globin-based carriers or gene upregulation in grafts.

HIF-1α, Hypoxia-inducible factor 1 alpha; PDK1, Pyruvate dehydrogenase kinase 1; LDH-A, Lactate dehydrogenase A; GLUT1, Glucose transporter 1; PDH, Pyruvate dehydrogenase; BNIP3, BCL2/adenovirus E1B 19 kDa interacting protein 3; PINK1, PTEN-induced kinase 1; Parkin, E3 ubiquitin-protein ligase Parkin; TFEB, Transcription factor EB; LC3, Microtubule-associated protein 1 light chain 3; MAP1LC3B, Microtubule-associated protein 1 light chain 3B; mtKeima, Mitochondria-targeted Keima; DRP1, Dynamin-related protein 1; MFN1, Mitofusin 1; MFN2, Mitofusin 2; GPx, Glutathione peroxidase; GPX4, Glutathione peroxidase 4; GSH, Reduced glutathione; GSSG, Oxidized glutathione; MDA, Malondialdehyde; 4-HNE, 4-hydroxynonenal; HSPA5, Heat shock protein family A (Hsp70) member 5; BiP, Binding immunoglobulin protein; GRP78, 78 kDa glucose-regulated protein; DDIT3, DNA damage-inducible transcript 3; CHOP, C/EBP homologous protein; XBP1, X-box binding protein 1; KATP channel, ATP-sensitive potassium channel; VEGF, Vascular endothelial growth factor; Notch, Notch receptor; Ngb, Neuroglobin; Cygb, Cytoglobin; MSC, Mesenchymal stem cell. Upward arrows indicate stimulation; downward arrows indicate inhibition; horizontal arrows indicate the sequence of effects of cellular and physiological pathways.

**Table 2:** Experimental and translational strategies to mitigate hypoxia in pancreatic islet transplantation with oxygen carriers, oxygen-generating biomaterials, preconditioning, mesenchymal stem cell–based support, encapsulation, surface modification, and extracellular matrix engineering.

Stage/Strategy	Intervention Molecules/Devices	Mechanistic Details (How Hypoxia Is Mitigated)	Key Benefits	Main Challenges	References
Pre-transplant hypoxia/hyperoxia	Controlled O_2_ tension (hypoxic vs. hyperoxic culture), cytoprotective agents	Tuning pre-culture O_2_ (e.g., 35–50% O_2_) to maintain viability while limiting oxidative damage; cytoprotective agents reduce ROS and apoptosis during isolation and culture	Maintains islet viability and function; reduces central necrosis in large islets	Narrow therapeutic window between hypoxia and hyperoxia; oxidative stress at very high pO_2_	[264,265,266]
Oxygen-generating and oxygen-carrying systems	OxySite (CaO_2_/calcium peroxide-containing alginate scaffolds); photosynthetic cyanobacteria (Synechococcus lividus); marine oxygen carriers (M101, M201); perfluorocarbon-based carriers	Local, sustained O_2_ release via hydrolysis of peroxides; light-driven photosynthesis; high-solubility synthetic O_2_ carriers that limit HIF-1α stabilization and apoptosis	Sustains aerobic metabolism, reduces inflammatory cytokines, improves graft viability and function; may lower required islet mass	Controlling O_2_ release kinetics; risk of hyperoxia and oxidative damage; device complexity and regulatory issues	[267,268,292]
Ischemic/hypoxia preconditioning	Intermittent hypoxia; brief non-lethal ischemia–reperfusion	Activates IL-6–Reg/HGF axis, pro-survival pathways, and mitochondrial targets; enhances β-cell proliferation and inhibits apoptosis; improves endothelial cell survival	Enhances β-cell function, insulin synthesis and secretion, and islet recovery after cold ischemia	Defining optimal duration/intensity; variable responses across species and islet preparations	[123,270,272,273,274,275]
Implantable oxygen transporters	Silicone/Parylene-based implantable oxygen transporter devices	Passive diffusion of atmospheric O_2_ through tubing to a subcutaneous islet-containing chamber, increasing local pO_2_ >120 mmHg during revascularization	Improves graft oxygenation and function in vivo without systemic O_2_ carriers	Surgical implantation, risk of infection, device durability and patient acceptability	[279,283]
MSC-based support	Mesenchymal stem cells (MSCs); MSC-conditioned media; MSC-derived exosomes	Paracrine secretion of VEGF, HGF, cytokines, ECM proteins and microRNAs; activation of PI3K/Akt, ERK1/2 and HIF-1α/PFKFB3 pathways; reduced oxidative stress, apoptosis and autophagy; enhanced angiogenesis	Improves islet viability and insulin secretion; enhances hypoxia tolerance and revascularization; enables cell-free “exosome” therapy	Standardizing MSC sources, dosing and preconditioning; long-term safety and manufacturing complexity	[282,283,284,293]
Islet encapsulation and surface engineering	Polymeric hydrogel microcapsules (e.g., alginate variants); amphiphilic PEG–bilirubin nanoparticles (BRNPs); Hep-PEG (heparinized starPEG nanocoating); oxygenated macroencapsulation devices with in situ O_2_ generation or electrolysis	Semi-permeable matrices allow nutrient/insulin diffusion but block immune cells; BRNPs scavenge ROS and suppress macrophage cytokine production; Hep-PEG nanofilms reduce inflammatory signalling and support islet viability; macrodevices integrate O_2_-generating biomaterials or continuous electrolysis-based O_2_ supply	Protects from immune attack and hypoxia; preserves insulin secretory function; can enable high-density islet packing with continuous oxygenation	Fibrotic overgrowth; ensuring uniform encapsulation and mass transport; large-scale manufacturing and long-term device retrieval	[268,286,287,294]
ECM and niche engineering	ECM proteins (Nidogen-1, Decorin); tailored hydrogel scaffolds	Restore β-cell–ECM interactions, upregulate glycolytic and pro-survival genes, reduce DNA fragmentation under hypoxia; support vascular ingrowth	Improves β-cell survival and function under hypoxic culture; may reduce required islet dose	Complexity of ECM composition; translation from in vitro to clinical-grade products	[295]

ROS, Reactive oxygen species; IL-6, Interleukin-6; HGF, Hepatocyte growth factor; MSCs, Mesenchymal stem cells; VEGF, Vascular endothelial growth factor; ECM, Extracellular matrix; PI3K, Phosphoinositide 3-kinase; ERK1/2, Extracellular signal-regulated kinases 1 and 2; PFKFB3, 6-phosphofructo-2-kinase/fructose-2,6-bisphosphatase 3; PEG, Polyethylene glycol; BRNPs, Bilirubin nanoparticles (PEG–bilirubin nanoparticles); Hep-PEG, Heparin-conjugated star-shaped polyethylene glycol nanocoating; NID1, Nidogen-1; DCN, Decorin.

## Data Availability

Data sharing not applicable to this article as no datasets were generated or analysed during the current study.
